# A longitudinal circulating tumor DNA-based model associated with survival in metastatic non-small-cell lung cancer

**DOI:** 10.1038/s41591-023-02226-6

**Published:** 2023-03-16

**Authors:** Zoe June F. Assaf, Wei Zou, Alexander D. Fine, Mark A. Socinski, Amanda Young, Doron Lipson, Jonathan F. Freidin, Mark Kennedy, Eliana Polisecki, Makoto Nishio, David Fabrizio, Geoffrey R. Oxnard, Craig Cummings, Anja Rode, Martin Reck, Namrata S. Patil, Mark Lee, David S. Shames, Katja Schulze

**Affiliations:** 1 Genentech Inc., South San Francisco, CA USA; 2 Foundation Medicine Inc., Cambridge, MA USA; 3 AdventHealth Cancer Institute, Orlando, FL USA; 4 The Cancer Institute Hospital, Japanese Foundation for Cancer Research, Tokyo, Japan; 5 F. Hoffman-La Roche AG, Basel, Switzerland; 6 LungenClinic Grosshansdorf, Airway Research Center North, German Center for Lung Research, Grosshansdorf, Germany

**Keywords:** Non-small-cell lung cancer, Tumour biomarkers, Clinical trial design, Machine learning

## Abstract

One of the great challenges in therapeutic oncology is determining who might achieve survival benefits from a particular therapy. Studies on longitudinal circulating tumor DNA (ctDNA) dynamics for the prediction of survival have generally been small or nonrandomized. We assessed ctDNA across 5 time points in 466 non-small-cell lung cancer (NSCLC) patients from the randomized phase 3 IMpower150 study comparing chemotherapy-immune checkpoint inhibitor (chemo-ICI) combinations and used machine learning to jointly model multiple ctDNA metrics to predict overall survival (OS). ctDNA assessments through cycle 3 day 1 of treatment enabled risk stratification of patients with stable disease (hazard ratio (HR) = 3.2 (2.0–5.3), *P* < 0.001; median 7.1 versus 22.3 months for high- versus low-intermediate risk) and with partial response (HR = 3.3 (1.7–6.4), *P* < 0.001; median 8.8 versus 28.6 months). The model also identified high-risk patients in an external validation cohort from the randomized phase 3 OAK study of ICI versus chemo in NSCLC (OS HR = 3.73 (1.83–7.60), *P* = 0.00012). Simulations of clinical trial scenarios employing our ctDNA model suggested that early ctDNA testing outperforms early radiographic imaging for predicting trial outcomes. Overall, measuring ctDNA dynamics during treatment can improve patient risk stratification and may allow early differentiation between competing therapies during clinical trials.

## Abstract (web summary)

A machine learning model that uses longitudinal ctDNA metrics robustly predicts survival in two phase 3 trials of patients with metastatic NSCLC, which may improve therapy selection and risk stratification.

## Main

One of the great challenges in therapeutic oncology is determining who might achieve survival benefits from a particular therapy. Cytotoxic agents, such as platinum-based alkylating agents or small-molecule inhibitors of receptor tyrosine kinases, can lead to observable reductions in overall tumor burden as measured by computerized tomography (CT) or magnetic resonance imaging. Imaging-based evaluation of the therapeutic effects of oncology drugs during the course of treatment informs on the response of a patient’s tumor to the drug or drug combination, prognosis of the patient and aids in physician decision making. In addition, imaging-based modalities have been developed as surrogates of overall survival (OS) and are widely used endpoints in oncology drug trials^1,2^. However, for certain types of drugs including cancer immunotherapies, progression-free survival (PFS) or overall response rate do not always correlate with OS^3,4^. Because of this lack of correlation between surrogate measures of drug efficacy and OS, oncology drug trials often depend on OS as a primary endpoint^5,6^. This means that trials can take many years to complete. Therefore, there is an important need to evaluate immunotherapy drug efficacy early in the course of therapy using alternative methods that are better associated with OS.

Circulating tumor DNA (ctDNA) testing has the potential to transform patient management by providing real-time assessments of patient prognoses and response to treatment using a simple blood draw^7^. ctDNA is a subset of the total cell-free DNA circulating in the bloodstream, thought to be shed by necrotic or apoptotic cells^8^. It can be profiled using next-generation sequencing as well as other methods and can be differentiated from background cell-free DNA by the presence of somatic tumor mutations^8^.

In the surgically resectable cancer setting, a positive ctDNA test after surgery has shown to be a poor-prognostic factor^9–14^ and changes in ctDNA correlate with treatment response^15–17^. In the metastatic setting, treatment response and survival times have been associated with changes in ctDNA levels during systemic treatment with chemotherapy^18,19^, targeted therapies^20,21^, immune checkpoint inhibitors (ICIs)^19,22^ and combination chemo-ICI^23^. The relatively higher ctDNA levels in patients with metastatic cancer compared to early-stage disease^24^ suggest this setting would be well suited for developing ctDNA as an early endpoint for new drug or combination evaluation, or to inform risk-based treatment decisions^25,26^.

The clinical implementation of ctDNA dynamics as a surrogate of survival has thus far been limited by small sample sizes, study designs without randomization or a lack of clarity on which ctDNA collection time points and summary metrics are optimal for predicting survival outcomes. To address these challenges, we performed high-sensitivity longitudinal ctDNA testing of 311 genes including correction for clonal hematopoiesis of indeterminate potential (CHIP) variants in 466 patients across 5 time points (1,954 samples total) in the phase 3 IMpower150 trial (NCT02366143).

The IMpower150 study was a randomized, open-label study that evaluated the safety and efficacy of anti-PD-L1 atezolizumab in combination with carboplatin + paclitaxel with or without bevacizumab compared with treatment with carboplatin + paclitaxel + bevacizumab in chemotherapy-naive participants with Stage IV nonsquamous non-small-cell lung cancer (NSCLC). The IMpower150 study met its primary endpoints of PFS (PFS hazard ratio (HR) = 0.62; 95% confidence interval (CI), 0.52–0.74; *P* < 0.001) and of OS (OS HR = 0.78; 95% CI, 0.64–0.96; *P* = 0.02), which led to the approval of atezolizumab + carboplatin + paclitaxel + bevacizumab in 1 L NSCLC^27^. Clinical data used in this work are based on the final OS analysis for the study (OS HR = 0.80; 95% CI, 0.67–0.95; data cutoff September 13, 2019)^28^. Atezolizumab is also an approved treatment in the early lung cancer setting^29^ as well as for other tumor types^30–34^.

After performing longitudinal ctDNA testing in IMpower150, we (1) examined the utility of individual ctDNA metrics to risk stratify patients including those with stable disease (SD) or partial responses (PR), (2) leveraged a machine learning approach in a training/testing framework to jointly model multiple ctDNA metrics to predict landmark survival, and (3) performed simulations to investigate whether our ctDNA model could outperform early radiographic imaging to detect differences between treatment arms in early clinical trial scenarios.

## Results

### Experimental plan and assay development

**Fig. 1:**
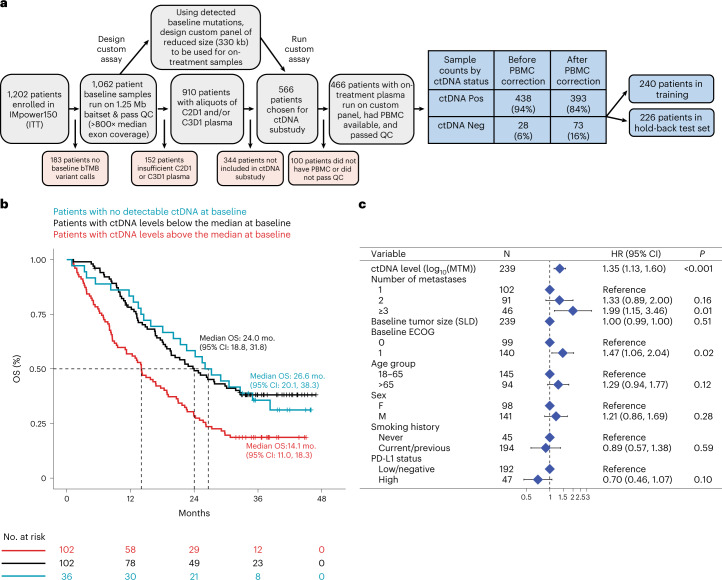
Design of ctDNA substudy and prognostic value of baseline ctDNA in training set. **a**, Consort diagram showing how the final 466 patients in the ctDNA evaluable population were identified and showing the prevalence of ctDNA positivity at the baseline time point before and after PBMC correction. **b**, Kaplan–Meier analysis showing the prognostic value of baseline ctDNA for OS in the training set of patients (*n* = 240), where blue curve indicates ctDNA negative patients (zero mutations detected), red curve indicates patients with ctDNA levels greater than or equal to the median (≥64 MTM) and black curve indicates patients with ctDNA levels less than the median. **c**, Multivariable Cox regression including baseline clinical features confirms that the ctDNA level is an independently poor prognostic factor for OS (*n* = 239 patients with nonmissing data available for all baseline clinical features). Two-sided Wald test *P* values are reported, and points and error bars indicate HR and 95% confidence interval, respectively. The exact *P* value for the first row ‘*P* < 0.001’ is 0.000672. MTM, mean tumor molecules.

Of the 1,202 patients enrolled in IMpower150, baseline plasma samples from 1,062 patients were evaluated using a prototype version of the FoundationOne Liquid CDx assay by Foundation Medicine Inc. (FMI), which sequenced >1.25 Mb of genomic content covering 394 genes^35^ (Fig. 1a). Sequence data were processed by a cell-free DNA computational pipeline that corrected errors via the use of fragment barcodes as previously described^35^. After the algorithmic removal of common germline and CHIP mutations, putative tumor-derived somatic alterations were identified at this baseline time point (Methods).

**Extended Data Fig. 1:**
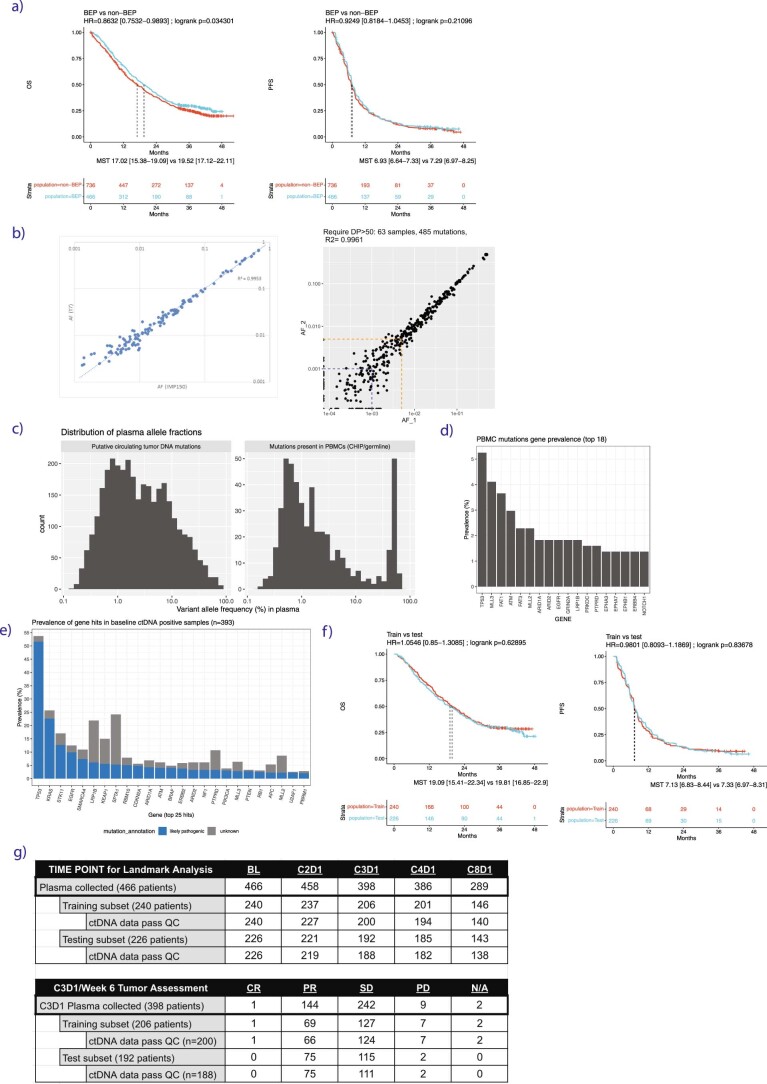
**(a)** KM curves showing OS (left) and PFS (right) for IMpower150 patients in the ctDNA biomarker evaluable population (BEP, blue) versus the ctDNA non-biomarker-evaluable-population (non-BEP, red). **(b)** Quality control experiments to show (left panel) high concordance of 330kb custom assay (‘IMP150’) compared to larger 1.25Mb assay (‘T7’), and to show (right panel) high reproducibility and sensitivity of 63 samples run in replicate on the 330kb custom assay where the LOD of the assay is found to be near 0.1% (where 85% of mutations near this frequency are detected reproducibly, blue dashed line) and the LOQ of the assay is near 0.5% (where the % CV of mutations near this frequency is 18%, orange dashed line). **(c)** Histogram of variant allele frequencies (%) for mutations identified using the custom 330kb panel, showing mutations present in plasma cell-free DNA and absent from PBMCs (left), and for mutations identified in plasma cell-free DNA and present in PBMCs (right). **(d)** Bar plot showing the genes in which PBMC-derived mutations (CHIP/germline) were most prevalent (*y* axis, percent of patients). PBMC-derived mutations are defined as those which were identified in both cell-free DNA and PBMCs for genes included in the custom 330kb panel. **(e)** Bar plot showing the genes in which tumor-derived mutations were most prevalent (*y* axis, percent of patients). Mutations that are known or likely pathogenic alterations (blue) are delineated from those which are variants of unknown significance (gray). Tumor-derived mutations are defined as those detected in cell-free DNA and absent from PBMCs for genes included in the custom 330kb panel. **(f)** KM curves showing OS (left) and PFS (right) for patients in the ctDNA biomarker evaluable population in the training split (blue) versus the test split of data (red). **(g)** Table showing the number of plasma samples collected at each time point, including a breakdown of the number in the training and test subsets which passed ctDNA assay QC, which were used for model training and testing. The bottom table shows number of patients who had C3D1 plasma samples which passed ctDNA assay QC and also treatment response assessments available for week 6 tumor assessment.

A subset of patients (*n* = 466) was chosen to be evaluated for on-treatment time points where we required the patient to have plasma available for C2D1 and/or C3D1, as well as peripheral blood mononuclear cells (PBMCs) available if putative tumor-derived variants were detected in baseline plasma (*n* = 438; Fig. 1a). Although common germline and CHIP variants were removed algorithmically in the original samples (see Methods), matched normal PBMCs were still required to remove less common germline and CHIP variants including those in canonical driver genes^36^. We expected a survivorship bias in the ctDNA-evaluable population due to our requirement for patients to have samples available after randomization, and while no strong PFS bias was found for ctDNA evaluable versus nonevaluable (HR = 0.92 (0.82–1.05)), we did detect a survivorship bias for OS (HR = 0.86 (0.75–0.99); Extended Data Fig. 1a). However, baseline characteristics were similar between the intention-to-treat (ITT) and the ctDNA evaluable population, including baseline Eastern Cooperative Oncology Group (ECOG), age, sex, race, region, among others (Supplementary Table 1).

On-treatment time points were assessed using a custom fixed panel assay to track changes in ctDNA in response to therapy. A custom assay was used to allow a higher depth of coverage for a similar cost. The final hybridization capture panel reduced the total panel size to 330 kb while capturing mutations in 311 genes present in ~94% of the IMpower150 patients’ baseline samples (Fig. 1a; Methods). Proprietary software developed by FMI was used to estimate on-treatment variant allele frequency (VAF) for every mutation detected at baseline (Methods). The final assay was experimentally validated to be highly concordant with the baseline assay and to have high sensitivities down to ~0.1% VAF (Extended Data Fig. 1b; Methods). The matched normal PBMCs were also run on this custom panel at high sequencing coverage (average mean target ~5,400× consensus deduplicated), and variants detected in both plasma and PBMCs were considered germline or CHIP mutations^37^.

There were 282 (64%) patients who had plasma variants that were also detected in PBMCs, including 45 patients who switched from ctDNA positive (at least one mutation detected) to ctDNA negative (zero mutations detected) after this PBMC correction (Fig. 1a). The number of PBMC-derived variants detected among these 282 patients ranged from 1 to 7, with mean 1.8 and median of 1 variant. The PBMC-derived germline/CHIP mutations had allele frequencies in plasma that overlapped with somatic tumor mutations in plasma (range 0.175–69% for PBMC-derived mutations and 0.14–82% for somatic tumor mutations, medians 1.3 and 2.2%, respectively; Extended Data Fig. 1c). Common CHIP genes were excluded from our custom panel (*TET2, DNMT3A, CBL, PPM1D, CHEK2, JAK2, ASXL1* and *SF3B1*). Among the 311 genes included in our panel, the PBMC-derived mutations were most prevalent in *TP53* (5.3% of patients), followed by *MLL3* (4.1%), *FAT1* (3.6%) and *ATM* (3.0%; Extended Data Fig. 1d). All PBMC-derived variants were subtracted from the final plasma mutation dataset.

**Fig. 2:**
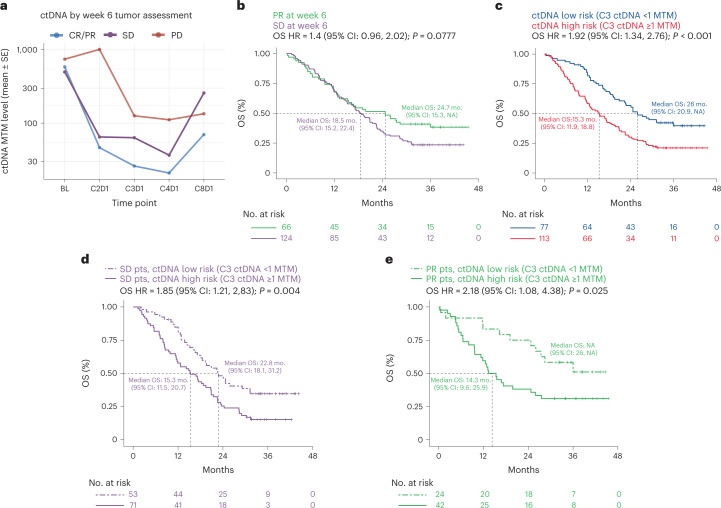
On treatment ctDNA dynamics associate with clinical outcomes in the training dataset. **a**, On-treatment ctDNA levels as measured by MTM (per milliliter plasma) across longitudinal time points for patients with week 6 radiographic assessments of treatment response of PD (red), SD (purple) and CR/PR (blue). **b**, KM curves showing OS for patients with SD (purple) versus PR (green) as determined at the week 6 radiographic assessment of treatment response. A univariable Cox proportional-hazards model was used to estimate HR and log-rank test to report *P* value. **c**, KM curves showing OS for patients with C3D1 ctDNA levels below the LOD of the assay (<1 MTM, ctDNA low risk, blue) versus near or above the LOD (≥1 MTM, ctDNA high risk, red). A univariable Cox proportional-hazards model was used to estimate HR and log-rank test to report *P* value. The exact *P* value for ‘*P* < 0.001’ is 0.00029871. **d**,**e**, KM curves showing OS for patients with SD (**d**) and PR (**e**) at week 6 who are further risk stratified by ctDNA levels at C3D1. A univariable Cox proportional-hazards model was used to estimate HR and log-rank test to report *P* value. MTM, mean tumor molecules.

**Fig. 3:**
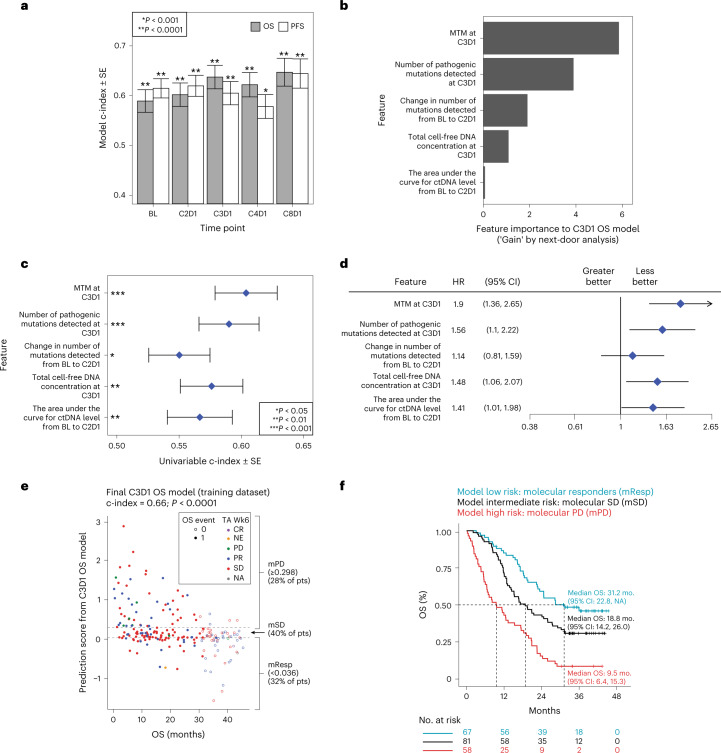
Building a machine learning model in the training dataset. **a**, Model performance for each survival outcome (PFS, OS) and plasma collection (BL thru C8D1) estimated by rank concordance (c-index) calculated from leave-one-out-cross-validation (LOOCV) to fit an elastic net model with ctDNA features. Bar height indicates c-index estimate, error bars indicate ± the standard error of the c-index, and two-sided *P* values are shown comparing each model’s c-index to random classifier. Each model is built using patients in the training subset at-risk for the relevant landmarked survival endpoint, where the numbers from left to right are: 240, 240, 237, 237, 206, 202, 201, 196, 146 and 136. The exact *P* values from left to right are 6.69 × 10^−5^, 9.50 × 10^−10^, 1.06 × 10^−5^, 7.97 × 10^−9^, 2.87 × 10^−9^, 4.16 × 10^−6^, 3.35 × 10^−7^, 0.000797098, 6.54 × 10^−8^, 3.18 × 10^−7^. **b**, Gain metric by next-door analysis for the five top features identified during LOOCV for the C3D1 OS ctDNA model. **c**, Univariable c-index showing the strength of association between OS from C3D1 (*n* = 206 patients) and each of the five top features for the C3D1 OS ctDNA model. Error bars indicate ± standard error of the c-index. Exact values from top to bottom for the two-sided *P* values comparing c-index to a random classifier are 2.23 × 10^−5^, 1.35 × 10^−4^, 0.0366, 0.0021 and 0.0093. **d**, Forest plot showing the HR for OS from C3D1 (*n* = 206 patients) estimated by univariable Cox proportional-hazards model, using the median value for the feature split, for the five top features for C3D1 OS ctDNA model. Higher feature values (above median) were generally associated with worse OS (HR above 1). Points and error bars indicate HR and 95% CI, respectively. **e**, Scatterplot showing final C3D1 OS ctDNA model predictions (*y* axis) versus OS time (*x* axis) in the training data, with dotted lines indicating the thresholds chosen in training set for mPD (≥0.298 prediction score), molecular response (mResp < 0.036) and molecular stable disease (mSD for (0.036, 0.298)). The exact value for the two-sided *P* value comparing the final C3D1 OS model’s c-index to a random classifier *P* value indicated by ‘*P* < 0.0001’ is 1.318316 × 10^−12^. **f**, KM curve showing that the final C3D1 OS ctDNA model can risk stratify patients in the training data.

**Fig. 4:**
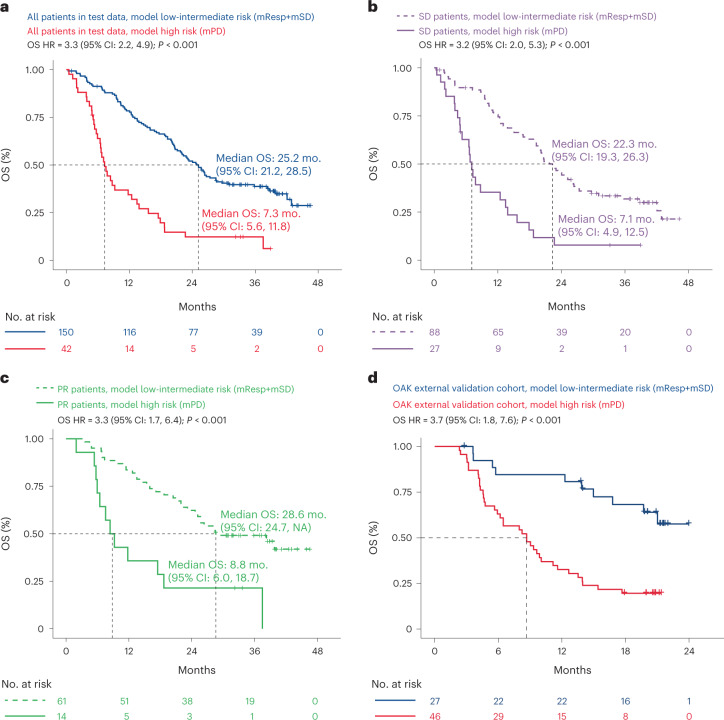
Machine learning model performs well for risk stratification in the hold-back test dataset and in the OAK external validation cohort. **a**, KM curve showing that the final C3D1 OS ctDNA model can be used for risk stratification in the hold-back test data, where patients with mPD (red) have worse OS compared to patients with a molecular response or molecular stable disease (mResp + mSD, blue). A univariable Cox proportional-hazards model was used to estimate HR and log-rank test to report *P* value. The exact *P* value indicated by ‘*P* < 0.001’ is 3.7228 × 10^−10^. **b**, KM curve showing that patients with radiographic treatment response of SD at the week 6 tumor assessment can be risk stratified using the final C3D1 OS ctDNA model in the hold-back test data, identifying SD/ctDNA high-risk patients (mPD, solid curve) and SD/ctDNA low-intermediate risk patients (mSD + mResp, dashed curve). A univariable Cox proportional-hazards model was used to estimate HR and log-rank test to report *P* value. The exact *P* value indicated by ‘*P* < 0.001’ is 8.8076 × 10^−7^. **c**, KM curve showing that patients with radiographic treatment response of PR at the week 6 tumor assessment can be risk stratified using the final C3D1 OS ctDNA model in the hold-back test data, identifying PR/ctDNA high-risk patients (mPD, solid curve) and PR/ctDNA low-intermediate risk patients (mSD + mResp, dashed curve). A univariable Cox proportional-hazards model was used to estimate HR and log-rank test to report *P* value. The exact *P* value indicated by ‘*P* < 0.001’ is 0.0003018. **d**, KM curve showing that the C3D1 OS model applied to the external validation cohort of 73 patients from the OAK clinical trial can provide predictions that identify high-risk patients in this 2nd line mNSCLC setting that used a distinct ctDNA assay technology. A univariable Cox proportional-hazards model was used to estimate HR and log-rank test to report *P* value. The exact *P* value indicated by ‘*P* < 0.001’ is 0.000119.

**Fig. 5:**
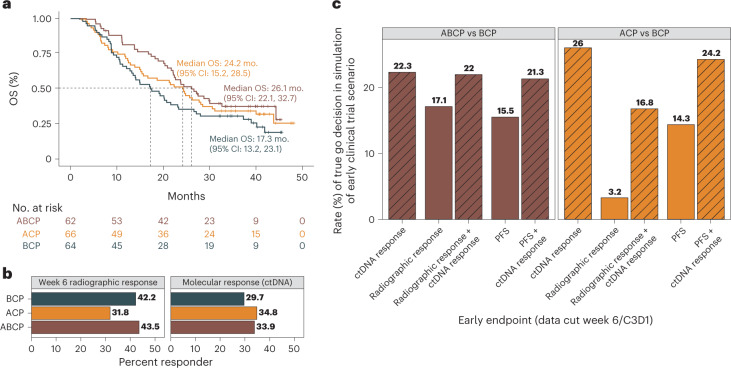
Machine learning model may be useful for detecting differences between treatment arms in early phase 2 clinical trial scenarios. **a**, KM curve showing OS in the test dataset for the three arms in the IMpower150 trial including ABCP (brown) versus ACP (orange) versus the control arm of BCP (black, control arm). **b**, Bar plot showing the rate of radiographic response at the week 6 tumor assessment for each treatment arm (left panel, CR/PR by RECIST criteria), and the rate of ctDNA molecular response for each treatment arm (right panel, mResp by C3D1 OS ctDNA model). **c**, Bar plot showing results from simulations of early phase 2 clinical trial scenario utilizing test data, where an early endpoint based on ctDNA (mResp by C3D1 OS model) is compared to early radiographic endpoints (week 6 RECIST response, week 6 PFS). Bar height corresponds to the proportion of simulations in which the active arm had higher rates of treatment response compared to control arm (‘true go rate’) for each early endpoint (*x* axis), where the left panel shows simulations comparing active ABCP arm to control BCP arm (left panel, brown colors), and right panel shows simulations comparing active ACP arm to control BCP arm (right panel, orange colors). *X* axis corresponds to which early endpoint is used in the simulation, comparing ctDNA criteria alone (mResp by C3D1 OS model), radiographic response alone (CR/PR by RECIST), PFS alone, or ctDNA added to radiographic response or PFS response.

ctDNA was detected in 393 patients (84%) at the baseline time point, of which 348 (89%) had pathogenic alterations detected including in the genes *TP53* (52%), *KRAS* (23%), *STK11* (13%) and *EGFR* (10%; Extended Data Fig. 1e). For downstream analyses, the ctDNA-evaluable population was split into a training (*n* = 240) and test set (*n* = 226; Fig. 1a), which were similar in survival outcomes (Extended Data Fig. 1f), baseline clinical features and ctDNA status (Supplementary Table 2). We noted that the number of patients for each treatment arm in training and test set was well balanced; atezolizumab + bevacizumab + carboplatin + paclitaxel (ABCP) 35% (*n* = 84) and 32.3% (*n* = 73) in training and test set, respectively, atezolizumab + carboplatin + paclitaxel (ACP) 31.2% (75) and 34.5% (78), and bevacizumab + carboplatin + paclitaxel (BCP) 33.8% (81) and 33.2% (75) (Supplementary Table 2). Final sample counts can be found in the supplement (Extended Data Fig. 1g). Note that all initial exploratory analyses and model building shown in Figs. 1–3 were performed in the training set of data, after which model validation was performed in the hold-back test set shown in Figs. 4 and 5.

### ctDNA levels are prognostic in training data

**Extended Data Fig. 2:**
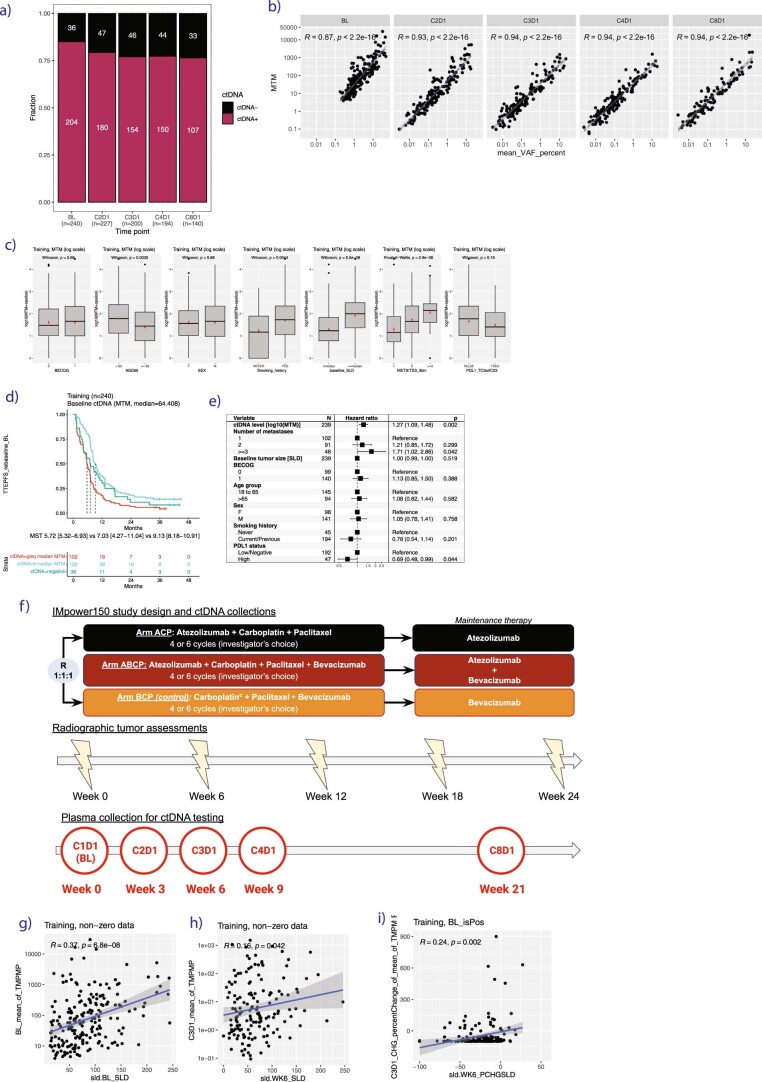
**(a)** Bar plot of number of ctDNA positive and negative samples at each time point in training. **(b)** Scatterplots showing correlation between mean variant allele frequencies versus mean tumor molecules per ml plasma in training. The baseline timepoint, in addition to having higher patient ctDNA tumor fractions due to occurring prior to treatment initiation, was sequenced with a 1.25Mb assay with reportable VAF range down to ~0.5%. On-treatment time points were sequenced with a 330kb assay with a reportable range down to ~0.01%, and restricted to only mutations detected at baseline. Pearson’s correlation coefficient is reported and its *P* value based on Pearson’s product moment correlation. **(c)** Boxplots showing association between baseline clinical features (6 panels, one for each feature) and ctDNA levels as measured by MTM (*y* axis) in training, where *P* values reported using a two-sided Wilcoxon rank sum test. The box plots depict the median at the middle line, with the lower and upper hinges at the first and third quartiles, respectively, the whiskers showing the minima to maxima no greater than 1.5× the interquartile range, and the remaining outlying data points plotted individually. Additionally, the mean and standard error are overlayed as red points. Sample sizes for the box plots from left to right are n = 99, 140; 133, 107; 98, 142; 45, 195; 120, 120; 102, 92, 46; 177, 63. **(d)** KM curve showing the prognostic value of baseline ctDNA MTM levels for PFS in training data. **(e)** Multivariable cox regression for PFS in training data. Two-sided Wald test *P* values are reported, and points and error bars indicate HR and 95% confidence interval, respectively. **(f)** Study schema showing when radiographic and plasma collections were performed in the treatment course. (g-h) Scatterplots showing association between radiographic assessment of tumor size by SLD measurement (*x* axis) versus ctDNA levels measure by MTM (*y* axis) for **(g)** baseline time point, **(h)** C3D1 time point, **(i)** change from BL to C3D1. Plots restrict to patients with ctDNA detected at baseline. Error band indicates 95% confidence interval. Pearson’s correlation coefficient is reported and its *P* value based on Pearson’s product moment correlation.

The baseline prevalence of ctDNA positivity (at least one mutation detected) was 85% in the training split of data (204/240), and prevalence decreased to 79.3% at C2D1, 77.0% at C3D1, 77.3% at C4D1 and 76.4% at C8D1 (Extended Data Fig. 2a). Baseline ctDNA was assessed for its prognostic value and association with baseline clinical features. Patients with any detectable ctDNA (*n* = 204) trended toward worse OS compared to the 36 ctDNA negative patients (HR = 1.33 (0.87–2.06), log-rank *P* = 0.19; median OS 18.8 versus 26.6 months from baseline, respectively). Among baseline ctDNA-positive patients, the median ctDNA level was 64 MTM (mean tumor molecules per ml plasma), which corresponded to a median of 1.4% mean allele frequency (AF). Note that MTM and mean AF were highly correlated (Extended Data Fig. 2b). Patients who were positive for ctDNA could be risk stratified using the median ctDNA level, where patients with ctDNA above the median had shorter OS compared to patients with ctDNA levels below the median (Fig. 1b; HR = 1.9 (1.36–2.64), log-rank *P* < 0.001; median OS 14.1 versus 24.0 months from baseline). Higher ctDNA levels were found to be associated with poor prognostic features including age below 65 years (Wilcoxon *P* = 0.0035), positive history of smoking (Wilcoxon *P* = 0.0044), baseline tumor size by the sum of longest diameters (SLD) above the median (Wilcoxon *P* < 0.001) and higher number of metastatic sites (Kruskal–Wallis *P* < 0.001; Extended Data Fig. 2c). However, the baseline ctDNA level was confirmed to be an independently poor prognostic factor for OS in a multivariable Cox regression model including baseline clinical features (HR = 1.35 (1.13–1.60), log-rank *P* < 0.001; Fig. 1c). Results were similar for PFS (Extended Data Fig. 2d,e).

Treatment initiation correlated with reductions in ctDNA levels, generally decreasing with each subsequent on-treatment time point through C4D1, which is the last time point in the series in which chemotherapy was given in combination with atezolizumab, bevacizumab or both (Extended Data Fig. 2f). Treatment responses as assessed by RECIST criteria at week 6 were associated with longitudinal ctDNA dynamics such that patients with CR or PR had lower ctDNA levels for all on-treatment ctDNA time points compared to patients with week 6 SD or progressive disease (PD; Fig. 2a). For example, baseline ctDNA-positive patients with a week 6 radiographic treatment response assessment of CR/PR (*n* = 67) tended to have greater reductions in ctDNA levels at C3D1 (mean −70% reduction in MTM level for CR/PR) compared to the 111 patients with week 6 SD (mean −39% reduction for SD) and the 16 patients with week 6 PD (mean +54% increase for PD; Kruskal–Wallis rank sum test *P* = 0.079). Radiographic tumor assessments were performed at baseline and every 6 weeks in the study, which is most contemporaneous with ctDNA collections at the BL and C3D1 (week 6) time points (Extended Data Fig. 2f). Levels of ctDNA were also correlated with tumor size (SLD) at BL and C3D1 (week 6; Pearson *R* = 0.37, *P* < 0.001 and Pearson *R* = 0.16, *P* = 0.042, respectively; Extended Data Fig. 2g,h). The percent change in ctDNA level from BL to C3D1 was correlated with the percent change in SLD from baseline to week 6 (Pearson *R* = 0.24, *P* = 0.002; Extended Data Fig. 2i).

Risk stratification using early radiographic tumor assessments alone showed numerically shorter OS for patients with week 6 SD compared to those with week 6 PR (median OS 18.5 versus 24.7 months; HR = 1.4 (0.96–2.02), log-rank *P* = 0.078; Fig. 2b). However, ctDNA data generated at a similar time point (C3D1) showed that patients who had ctDNA levels near or above the limit of detection (LOD) of the assay (≥1 MTM) had shorter OS compared to patients who maintained or reduced ctDNA to below the LOD (HR = 1.92 (1.34–2.76), log-rank *P* < 0.001; median 15.3 versus 26.0 months from C3D1 for patients with <1 and ≥1 MTM at C3D1, respectively; Fig. 2c).

**Extended Data Fig. 3:**
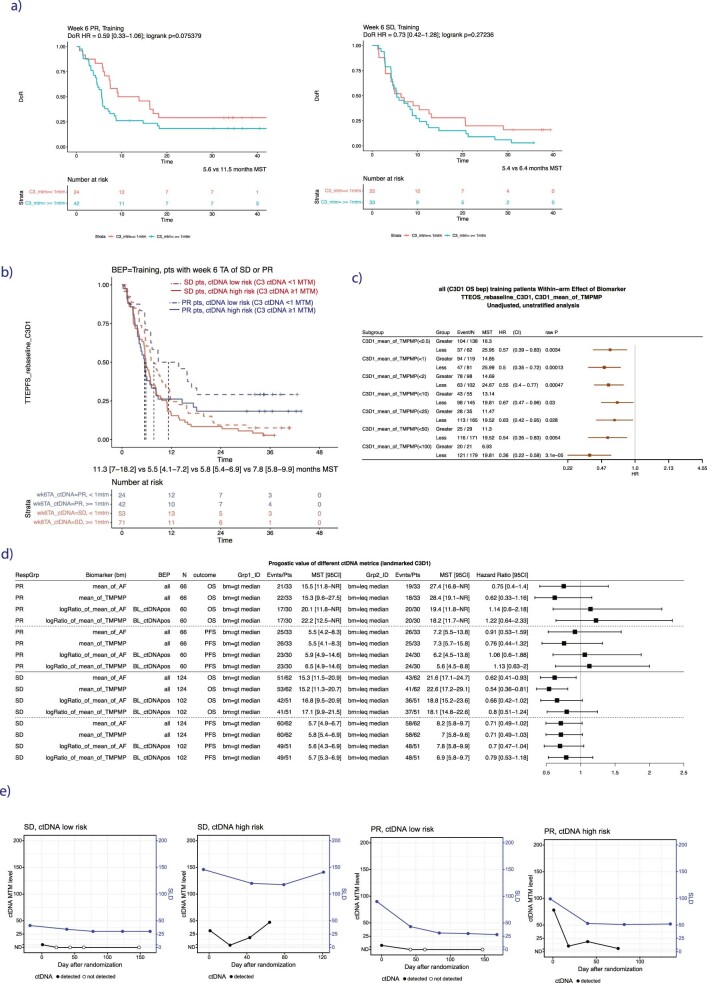
**(a)** KM analysis for duration of treatment response (DoR) in patients with PR (left) or SD (right) at week 6 tumor assessment who are risk stratified using ctDNA levels above or below 1 MTM, in training. **(b)** KM analysis for PFS in patients with SD or PR at week 6 tumor assessment who are risk stratified using ctDNA levels above or below 1 MTM, in training. **(c)** Forest plot showing prognostic value of other thresholds of MTM splits at C3D1 timepoint for risk stratification for OS in entire training dataset. Note that here MTM is labeled mean_of_TMPMP (for mean tumor molecules per ml plasma). HRs are comparing patients with MTM level below (‘Less’) versus above (‘Greater’) each threshold for splitting C3D1 MTM, where the number of patients can be found in the third column (‘N’). MST indicates median survival time. Points and error bars indicate HR and 95% confidence interval, respectively. Univariable Cox proportional-hazards model was used to estimate HR and logrank test to report *P* values. **(d)** Forest plot showing prognostic value of other ctDNA metrics for OS and PFS in PR and SD patients in training. Note that here MTM is labeled mean_of_TMPMP (for mean tumor molecules per ml plasma). HRs are comparing patients with feature values ≤ versus > than the median value for that feature. RespGrp column indicates whether the subset for the risk stratification analysis is the PR or SD patients. BEP column indicates ‘biomarker evaluable population’, meaning the subset of patients included in the analysis, which is either ‘all’ patients (for features summarizing ctDNA levels), or for patients who are ctDNA positive at the baseline time point (‘BL_ctDNApos’, for features summarizing ctDNA change). Outcome column indicates if the HR is for OS or PFS. MST indicates median survival time. Points and error bars indicate HR and 95% confidence interval, respectively. **(e)** Four example patient time courses showing longitudinal ctDNA MTM level and tumor size by SLD for 4 example patients.

We found that combining C3D1 ctDNA risk (≥1 versus <1 MTM) with week 6 treatment response by RECIST improved risk stratification further such that SD patients could be split into SD/ctDNA high-risk versus SD/ctDNA low-risk (Fig. 2d; OS HR = 1.85 (1.21–2.83), log-rank *P* = 0.004; median 15.3 versus 22.8 months from C3D1, respectively). Week 6 PR patients could also be split into PR/ctDNA high-risk versus PR/ctDNA low-risk (Fig. 2e; OS HR = 2.18 (1.08–4.38), log-rank *P* = 0.025; median 14.3 months from C3D1 versus median not reached, respectively). The week 6 PR patients who were high risk by ctDNA had numerically shorter duration of treatment response compared to patients who were low risk by ctDNA (median 5.6 versus 11.5 months duration of treatment response; duration of treatment response (DoR) HR = 0.59 (0.33–1.06), log-rank *P* = 0.075; Extended Data Fig. 3a). Results were similar when analyses were repeated using PFS (Extended Data Fig. 3b) or alternative ctDNA thresholds or metrics (Extended Data Fig. 3c,d). Visualizing the SLD dynamics parallel to ctDNA dynamics revealed that while ctDNA changes mirror SLD changes, patients with different response categories by radiographic imaging could still have very similar ctDNA patterns (Extended Data Fig. 2e). Overall, these results suggest that imaging-based risk stratification at early on-treatment time points may be improved upon by combining radiographic imaging with ctDNA measurements, potentially informing risk-based treatment decisions made by clinicians.

### Machine learning model predicts survival using ctDNA metrics

**Extended Data Fig. 4:**
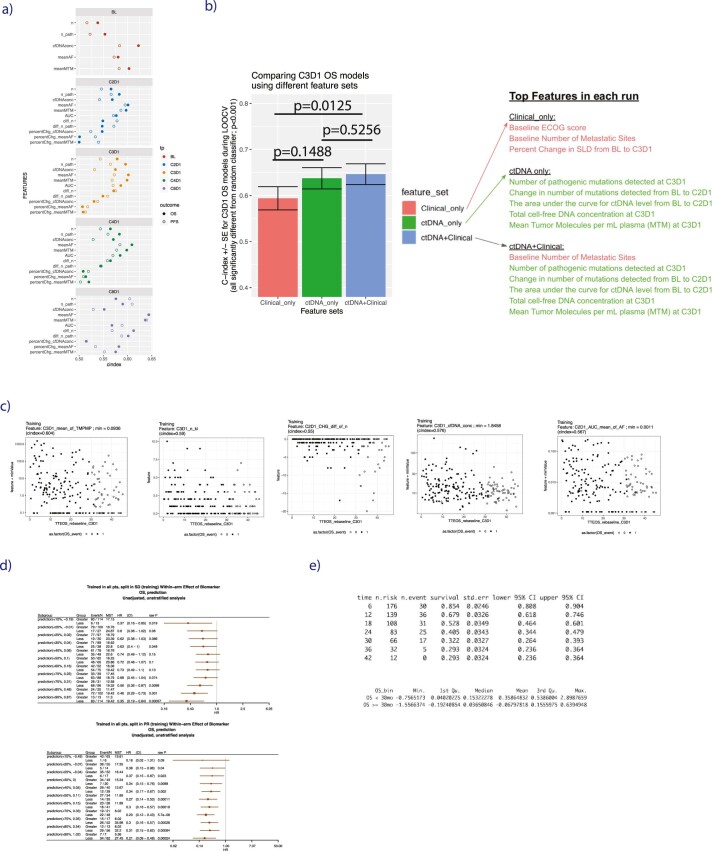
**(a)** Scatterplots showing the univariable rank concordance (c-index, *x* axis) for each individual ctDNA feature for landmarked OS and PFS estimated at each time point (panels), in training. ‘n’ indicates number of detected variants’, ‘n_path’ indicates number of detected known/likely pathogenic variants, ‘percChg’ and ‘diff’ indicate percent change and difference in ctDNA level from baseline. **(b)** Comparison of models trained using either clinical features alone (red), ctDNA features alone (green), or ctDNA+Clinical features (blue), with annotation as to which metrics were top features in each run. The bar height is rank concordance (c-index) calculated from leave-one-out-cross-validation (LOOCV) to fit an elastic net model, error bars are standard error of the c-index, *P* values are two-sided and based on a U-statistic to compare two predictors. Models were built using *n* = 206 patients in the training subset at-risk for an OS event at C3D1. **(c)** Scatterplots for the 5 top features from C3D1 OS model, showing the association between each feature value with landmark OS. **(d)** Forest plots showing prognostic value of C3D1 OS ctDNA model predictions in training data for patients with SD (top forest plot) and Partial Response (bottom forest plot), where the number of patients can be found in the third column (‘N’), ‘MST’ indicates median survival time. Points and error bars indicate HR and 95% confidence interval, respectively. Univariable Cox proportional-hazards model was used to estimate HR and logrank test to report *P* values. Note that the threshold chosen for categorizing a patient as having molecular progressive disease (mPD) was done by taking the mean of the optimal split in SD patients (75th percentile, top forest plot) and the optimal split in PR patients (70th percentile, bottom forest plot). **(e)** Choosing a threshold in training data of C3D1 OS ctDNA model predictions for categorizing a patient as having mResp was done by identifying the patients in training data who achieved durable OS of ≥ 30 months (which was 32.2% of the population, see top table), and then taking the median prediction score of this population (which was 0.036, see bottom table) in training data.

Multiple metrics can be used to describe ctDNA dynamics, for example, the ctDNA level can be measured using the AF, the MTM per milliliter plasma, the total cell-free DNA concentration, the number of mutations detected or the number of pathogenic mutations detected, among others^38^. We derived 19 metrics of the ctDNA level (measured for every time point) and 59 metrics of ctDNA change relative to baseline (measured for each on-treatment time point) (Supplementary Table 3; Methods). The performance of different metrics to individually predict landmark survival can be summarized using the rank concordance (Harrell’s c-index), and we find that performance varies across metric types, time points and survival outcomes in the training data (*n* = 240; Extended Data Fig. 4a). We next jointly modeled these features in a machine learning framework using an elastic net approach^39^ to predict landmark PFS and landmark OS from each plasma collection time point (see Methods). This permits many features related to ctDNA levels and changes to be included in the model initially; however, nested cross-validations during the training reduce the number of features to an optimal subset that minimizes prediction error.

Models were trained for each visit time point, where all measurements collected from baseline up until that particular visit were included as features, for example for C3D1 models, features from BL, C2D1 and C3D1 were included. Note that treatment arms were pooled to build a model useful as an early endpoint regardless of the treatment regime being used (similar concept to RECIST criteria). Pooling was also appealing because building a multivariable predictive model requires relatively large sample sizes in the training and testing splits. Results showed that model performance was generally best (higher c-index) for OS at the C3D1/C8D1 time points (Fig. 3a and Supplementary Table 4). We decided to focus on the OS model for the C3D1 time point given that C8D1 samples were available for only 60% of patients (which itself is a good prognostic/favored by immortal bias) and because OS is the most relevant metric for oncology therapeutics.

To investigate how a ctDNA-focused model compared to known prognostic factors, we compared C3D1 OS models trained using either clinical features alone or combined with ctDNA features. For clinical features, we included the baseline factors shown in Fig. 1c as well as measures of tumor size from early radiographic assessments at baseline and week 6 (see Methods). We find that when comparing C3D1 OS runs, the combined ctDNA + clinical feature set performs substantially better compared to the clinical features alone (*P* = 0.0125; Extended Data Fig. 4b and Supplementary Table 4).

To identify top features for the C3D1 OS ctDNA model, we chose ctDNA features that were chosen during training in >50% of cross-validations and which had a positive gain metric by next-door analysis^40^. This reduced the number of features in the model to just five metrics, which included metrics related to MTM, the number of detected mutations and the total cell-free DNA concentration (Fig. 3b, Supplementary Table 5). Individually, these metrics showed univariable c-indices for OS between 0.55 and 0.60 (Fig. 3c, Extended Data Fig. 4c and Supplementary Table 5), and the median split of each metric separately provided OS HRs between 1.14 and 1.90 (Fig. 3d).

The final C3D1 OS ctDNA model was fit using the five top ctDNA features in the entire training dataset with a final c-index of 0.66 (*P* < 0.001 for comparison to random classifier; Fig. 3e and Supplementary Table 6). Numeric thresholds for binning C3D1 OS model predictions were chosen in the training data (see Methods) to bin patients into three groups including those at high risk (molecular progressive disease (mPD), 28% of training set), those at low risk (molecular response (mResp), 32% of training set) and those with intermediate risk (molecular stable disease (mSD), 40% of training set; Fig. 3e, Extended Data Fig. 4d,e). Kaplan–Meier analysis confirmed the prognostic value for OS of these three bins in the training data (median OS from C3D1 of 9.5, 18.8 and 31.2 months for mPD, mSD and mResp, respectively; Fig. 3f).

**Extended Data Fig. 5:**
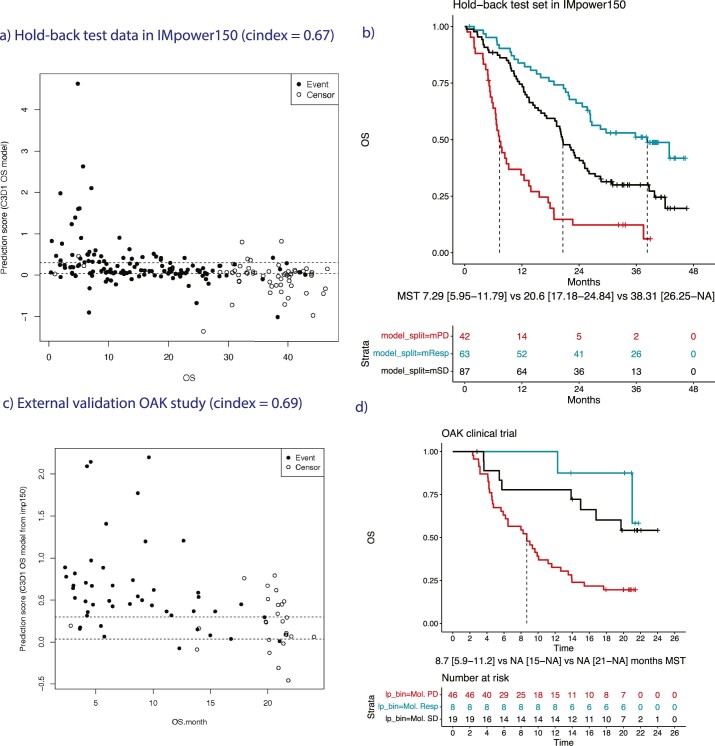
**(a)** Scatterplot showing the final C3D1 OS ctDNA model predictions (*y* axis) versus OS time (*x* axis) in the hold-back test data for IMpower150 (c-index, 0.67). Dotted lines show thresholds for mPD (≥ 0.298 prediction score), mResp (< 0.036 prediction score), and mSD (for [0.036, 0.298) prediction scores), which were thresholds chosen in the training set of data. **(b)** KM curves for OS in hold-back test set showing the final subgroups identified using the C3D1 OS model prediction thresholds chosen in training data. Subgroups include mPD (red line), mResp (blue line), and mSD (black line), all confirmed to have prognostic value in this test data. **(c)** Scatterplot showing the final C3D1 OS ctDNA model predictions (*y* axis) versus OS time (*x* axis) in the external validation OAK cohort of 73 patients (c-index, 0.69). Dotted lines show thresholds for mPD (≥ 0.298 prediction score), mResp (< 0.036 prediction score), and mSD (for [0.036, 0.298) prediction scores), which were thresholds chosen in the training set of data. **(d)** KM curves for OS in external validation OAK cohort of 73 patients showing the final subgroups identified using the C3D1 OS model prediction thresholds chosen in training data. Subgroups include mPD (red line), mResp (blue line), and mSD (black line), all confirmed to have prognostic value in this external validation data.

We tested the final C3D1 OS ctDNA model at a c-index of 0.67 (*P* < 0.001, Extended Data Fig. 5a) in the hold-back test set with evaluable C3D1 plasma (*n* = 192 patients, Fig. 1b) and found that the prespecified thresholds chosen in the training set also provided good separation between risk groups in the test data (Extended Data Fig. 5b). The ctDNA model high-risk group showed shorter OS compared to patients with low-intermediate risk (OS HR = 3.28 (2.2–4.9), log-rank *P* < 0.001; median 7.3 months versus 25.2 months from C3D1 for mPD and mResp + mSD, respectively; Fig. 4a). Model predictions in the test set also performed well in patients with a week 6 tumor assessment of SD (c-index in SD = 0.68; *P* < 0.001), and the prespecified threshold could risk stratify SD patients into high risk versus low-intermediate risk (OS HR in SD = 3.23 (1.98–5.29)], log-rank *P* < 0.001; median 7.1 versus 22.3 from C3D1 for mPD and mResp + mSD, respectively) (Fig. 4b). Similarly, ctDNA model predictions in the test set performed well in patients with PR (c-index in PR = 0.64; *P* = 0.002) and was able to identify a high-risk PR subgroup (OS HR in PR = 3.26 (1.66–6.42)), log-rank *P* < 0.001; median 8.8 versus 28.6 from C3D1 for mPD and mResp + mSD, respectively; Fig. 4c).

We further validated our C3D1 OS ctDNA model in an external patient cohort (*n* = 73), which is a subset of patients from the OAK clinical trial (NCT02008227) who had ctDNA assessed using the Avenio ctDNA assay at BL, C2D1 and C3D1^19^. In addition to a different ctDNA assay, the external cohort also included metastatic NSCLC patients from the 2nd line setting (versus 1st line in IMpower150) and different treatment regimes (monotherapy of atezolizumab or docetaxel versus chemo-ICI combinations in IMpower150). Applying our C3D1 OS predictor developed in the IMpower150 data to the OAK data (Methods), we find it validates well in this external cohort with a c-index of 0.69 (*P* < 0.0001; Extended Data Fig. 5c), and the prespecified cutoffs were also able to identify high risk (mPD) versus low-intermediate risk (mResp + mSD) patients in the OAK clinical trial (OS HR = 3.73 (1.83–7.60), log-rank *P* = 0.00012; Fig. 4d and Extended Data Fig. 5d). These findings support the potential clinical utility of our ctDNA model across multiple treatments and settings in NSCLC.

### ctDNA model utility as an early endpoint in drug development

**Extended Data Fig. 6:**
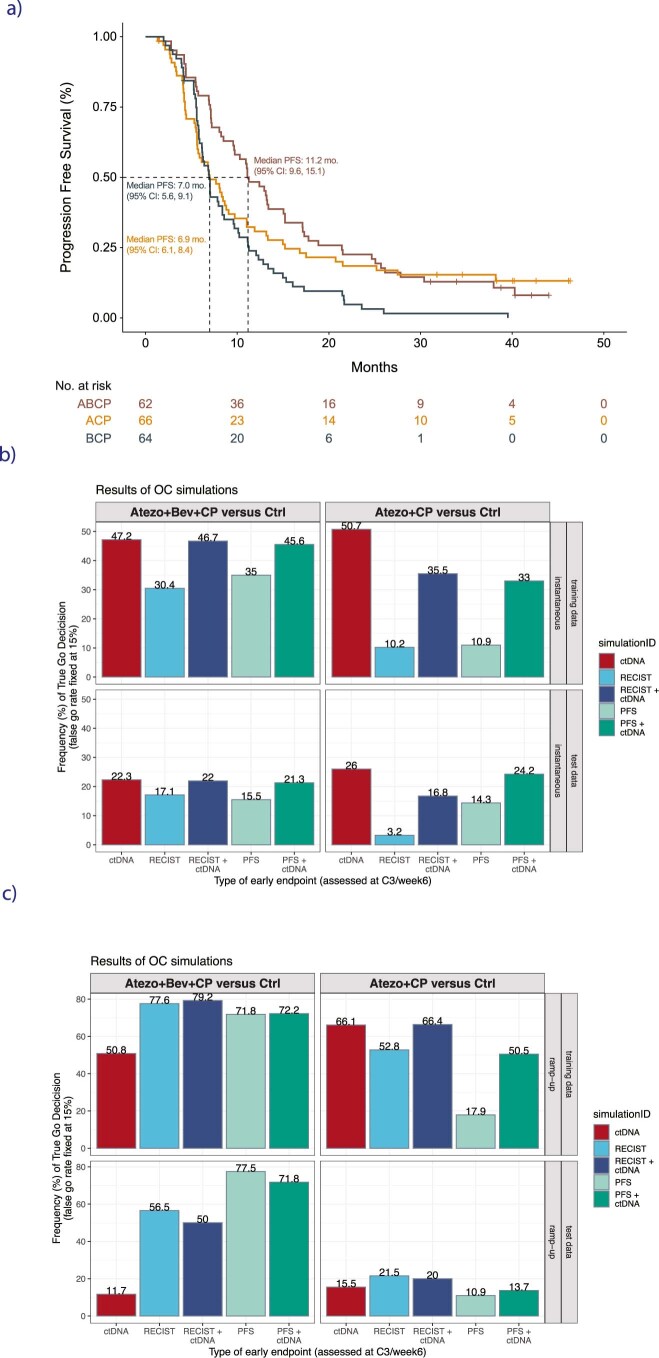
**(a)** KM curve showing PFS in the test dataset for the three arms in the IMpower150 trial including ABCP (brown), ACP (orange), and control arm BCP (black, control arm). (**b**–**c**) Complete results of operation characteristics simulations showing the rate of true ‘Go’ decisions in **(b)** instantaneous enrollment scenario (every patient has their clinical data cut at their respective C3D1 time point), versus **(c)** ramp-up enrollment scenario (use all clinical data available for patient after last patient enrolls, so some patients could have additional radiographic data available after the week 6 time point). Training data shown in top rows, test data shown in bottom rows. The early endpoint is either ctDNA criteria alone (red bar), RECIST criteria alone (light blue) or RECIST criteria combined with ctDNA (dark blue), PFS alone (light green bar) or PFS combined with ctDNA criteria (dark green bar).

For a new early endpoint to be useful for clinical drug development in metastatic lung cancer, it should sensitively detect differences in OS between treatment arms at an early time point. Additionally, it should improve upon more traditional early radiographic-based endpoints like RECIST response or PFS. In this IMpower150 study, patients in the control arm received BCP and patients in the experimental arms received either ABCP or ACP (Fig. 5a, Extended Data Fig. 6a, test data shown). We note that the US approval was based on comparing ABCP arm to BCP arm^27,41^, although the ABCP and ACP arms had similar OS results^28^. To explore whether our ctDNA C3D1 OS model could provide early signals of treatment efficacy, we compared rates of ctDNA mResp between the different treatment arms and contrasted with RECIST response and PFS assessed near week 6.

The rate of ctDNA mResp was 33.9% in ABCP and 34.8% in ACP versus 29.7% in BCP control arm (Fig. 5b). Whereas in week 6 the RECIST response rates were numerically higher for the active ABCP versus control BCP arms (43.5% in ABCP and 42.2% in BCP), the active ACP arm had a lower rate of radiographic response compared to control at this early week 6 time point (31.8% in ACP).

To quantify the utility of ctDNA as an early endpoint capable of informing drug development decision-making in an early phase 2 clinical trial scenario, we resampled IMpower150 to *n* = 30 patients per arm in *n* = 2,000 simulations using our test data. We then measured the proportion of simulations in which the active arm had higher rates of treatment response compared to control arm (‘true go rate’) using different early endpoints assessed near week 6 including ctDNA response, RECIST response, PFS, or a combination (see Methods section on operation characteristics simulations^42^). The results of this analysis showed that ctDNA response by itself had higher true go rates than either week 6 RECIST or PFS (Fig. 5c), suggesting that ctDNA response may be more useful than early radiographic assessments for detecting signals of drug efficacy. Additionally, combining the ctDNA response metric with RECIST improved the true go rate compared to RECIST by itself, as did combining ctDNA response metric with PFS (Fig. 5c). For completeness, we repeated the simulations in the training data and results were similar (Extended Data Fig. 6b), and we also modified simulations to use a ramp-up enrollment approach (see Methods) which showed the ctDNA response endpoint to have less utility (Extended Data Fig. 6c). Overall, these findings suggest that on-treatment ctDNA measurements may have utility as an early endpoint to support early decision-making in clinical trial scenarios.

## Discussion

To our knowledge, this is the first study to systematically evaluate the utility of longitudinal ctDNA dynamics across a large, randomized phase 3 clinical trial. We show that ctDNA metrics collected across longitudinal time points can be used to risk stratify patients and predict survival in patients with metastatic nonsquamous NSCLC treated with chemo-immunotherapy combinations. In addition to the high prognostic value of baseline ctDNA levels for PFS and OS, on-treatment ctDNA changes are correlated with treatment response and can be combined with radiographic imaging to provide finer risk stratification of patients who achieve PR or SD. Notably, in an external cohort of patients our model predicted high-risk patients despite differences in treatment setting and ctDNA technology, further supporting the potential clinical utility of ctDNA for predicting OS for immunotherapy and immunotherapy combinations in multiple NSCLC settings.

There are diverse approaches used in the literature to summarize ctDNA levels and integrate ctDNA features for association with clinical outcomes, as well as the open question regarding which on-treatment time points may be optimal for longitudinal ctDNA analyses^19,38,43,44^. We leveraged a machine learning approach to address these questions by jointly modeling multiple ctDNA metrics to predict landmark OS and PFS. We found C3D1 to be a preferred time point for model performance, and due to occur relatively early in the treatment timeline before some patients have disease progression. Additionally, we found top ctDNA features to include metrics related to the number of detected variants, the MTM per milliliter plasma and the total cell-free DNA extracted from plasma.

We also explored the utility of ctDNA as an early endpoint for detecting differences between treatment and control arms in randomized clinical trial settings. Using our final C3D1 OS model, we compared ctDNA response to radiographic endpoints assessed at week 6 including PFS and RECIST response. We found that ctDNA response outperforms RECIST and PFS, particularly when evaluating drugs that are not cytotoxic agents like immunotherapies. mResp by ctDNA could be very useful in early clinical development decision-making for these types of drugs.

In terms of study limitations, the number of patients with samples available decreased with each consecutive time point due to coming off the study at disease progression, potentially limiting the statistical power of our approach at later time points. We note that an early endpoint will be of limited utility for patients that progress so rapidly because their clinical outcomes are also apparent early in the study timeline. Another caveat of our work is that while we assessed the association of ctDNA with clinical benefit for chemo-ICI combinations as well as monotherapies, additional data should be generated in other treatment contexts (for example, targeted therapies) to delineate treatment-dependent ctDNA dynamics. Additionally, we focus on the association of ctDNA with OS which is an endpoint that can be confounded by poststudy treatment, and it is unknown how this may have affected our results. Lastly, this study used data from two separate ctDNA assays, both of which were panel-based approaches with high sensitivity down to ~0.1% ctDNA fraction and high specificity via PBMC correction. However, there are other assays currently available on the market and it is unclear whether assay choice may affect the correlation between ctDNA metrics and clinical outcomes. While we included a comparison of ctDNA features with baseline clinical factors, an interesting extension of our work would be to incorporate other on-treatment circulating biomarkers and assess whether they are superior to ctDNA for predicting outcomes.

Overall, we have mapped ctDNA dynamics in a large, randomized study with unprecedented resolution. We have shown that changes in ctDNA, as modeled in a machine learning framework and validated in both a hold-back test set and an external cohort, can improve patient risk stratification, as well as sensitively detect differences between treatment arms at early time points in clinical trial settings. ctDNA shows promise as an early endpoint for decision-making during drug development and, with prospective validation, potentially as a risk stratification tool to inform treatment decisions.

## Methods

### IMpower150 trial design, participants and endpoints

Details on the IMpower150 study plan and results have been published elsewhere^27^, and the study Protocol and Statistical Analysis Plan can be found on clinicaltirals.gov (NCT02366143). The protocol for IMpower150 was approved by ethics committees at each site, and the study followed the International Conference on Harmonisation Good Clinical Practice guidelines and accorded with the principles of the Declaration of Helsinki. The list of 161 ethics committees can be found in the reporting summary. All patients provided written informed consent and were not compensated for participation.

IMpower150 was a phase 3 study that randomly assigned patients in a 1:1:1 ratio to receive every 3 weeks either ACP group, or ABCP group or BCP group. Patients underwent radiographic (CT scan) tumor assessments until the occurrence of disease progression (according to RECIST v1.1 criteria) or until the loss of clinical benefit among patients who continued to receive atezolizumab after the initial disease progression. These assessments were performed at screening and every 6 weeks from cycle 1 day 1 for the first 48 weeks, and every 9 weeks thereafter. The primary endpoints were PFS (as assessed by investigators according to RECIST criteria) and OS. The final analysis including distribution of poststudy treatment usage has been reported previously^28^, although we note there is a lot of missingness in the poststudy treatment data due to patients coming off-study upon deviation from protocol-specified anticancer therapy.

Patient inclusion criteria were the following: stage IV or recurrent metastatic nonsquamous NSCLC without previously receiving chemotherapy, a ECOG performance-status score of 0 or 1 at baseline, available tumor tissue for testing and eligibility to receive bevacizumab. Patients who had received previous adjuvant or neoadjuvant chemotherapy were eligible if the last treatment was at least 6 months before randomization. Any PD-L1 immunohistochemistry status was eligible, and tumor PD-L1 expression (on tumor cells or tumor-infiltrating immune cells) was assessed in archival or freshly collected tissue (or both) with a PD-L1 immunohistochemistry assay (Ventana Medical Systems; clone SP142; N/A; predilute ready to use antibody product at 36 μg/5 ml). Patients having *EGFR* or *ALK* genomic alterations were included if they previously had treatment with 1+ approved tyrosine kinase inhibitor but had disease progression or unacceptable side effects. Exclusion criteria were untreated metastases of the central nervous system, autoimmune disease, or receiving previous immunotherapy or anti-CTLA-4 therapy within 6 weeks before randomization, or receiving systemic immunosuppressive medications within 2 weeks before randomization.

### Sample collection and processing, assay development and splitting into training/testing sets

The PBMCs used for analysis were isolated from one 8.5 ml of whole blood collected in an acid citrate dextrose tube at a specialty vendor and from an 8 ml cell preparation tube containing sodium citrate. The plasma used for analysis was separated from two times 6 ml of whole blood collected in K2 EDTA vacutainers and was processed within 30 min after blood collection.

Baseline plasma samples from 1,062 patients were retrospectively analyzed using the assay method described previously^35^.

On-treatment samples from 566 patients (C2D1, C3D1, C4D1 or C8D1) were evaluated with a custom 330 kb assay targeting 311 genes. The hybrid capture panel for this assay was designed by pooling the alterations found for all the samples in the baseline assay, filtering for known germline variants based on ExAC database (http://exac.broadinstitute.org/), known CHIP genes *TET2*, *DNMT3A*, *CBL*, *PPM1D*, *CHEK2*, *JAK2*, *ASXL1*, *SF3B1*, noncoding variants and repetitive regions, and <100× coverage. The resulting pool of alterations was clustered based on proximity within the genome, and clusters with four or more alterations plus smaller clusters that represented samples with less than three alterations were chosen for hybrid capture bait designs. The genomic regions of the clusters were compared to the baits in the baseline bait set, the corresponding baits were selected as the custom assay bait set.

The sequencing libraries were prepared using the same plasma extraction, library construction and hybrid capture-based methodology as FoundationACT with consistent analytical performance (that is, sensitivity, specificity) and has been previously described^35,45^. Briefly, between 1 ml and 5 ml of frozen plasma from each patient was sent to FMI. Once received, cfDNA was extracted, and isolated cfDNA was quantified using the 4,200 TapeStation (Agilent Technologies). A minimum of 20 ng of extracted cfDNA was required for a sample to undergo sequencing.

In this study, the assay LOD and lower limit of quantitation (LOQ) were determined to be 0.1% and 0.5%, respectively, as follows: 63 on-treatment samples with residual plasma were rerun through the sequencing library prep, construction, hybrid capture and sequencing pipeline. Based on the baseline time point, there was a total of 485 variants expected to be present in these samples. The AF of these 485 variants was then assessed in the replicate on-treatment samples (Extended Data Fig. 1b, right panel). The LOD of the assay, commonly defined as the lowest concentration of an analyte in a sample that can be consistently detected with a stated probability (typically near 90%), was then determined. It was found that the 32 variants with allele frequencies near 0.1% were detected with 85% probability across replicates. The LOQ of the assay, commonly defined as the lowest standard concentration that can be quantified with a % coefficient of variation (CV) value below a certain threshold (typically ~20–30%), was then determined. It was found that the 28 variants with allele frequencies near 0.5% had % CV of 18%. Therefore, the LOD and LOQ have been reported to be near ~0.1% and ~0.5%, respectively.

Matched whole blood or PBMC samples were sequenced to subtract germline and CHIP mutations. A total of 300 µl of sample was extracted using KingFisher platform (Thermo Fisher Scientific). The genomic DNA was sheared by ultrasonication to generate approximately 200-bp fragments (Covaris). Postshearing, 200 ng was used with the same protocol as the cfDNA samples mentioned above.

The training and test sets were initially chosen based on the sequencing batch for the set of 566 patients chosen for the ctDNA substudy (see Fig. 1a), where we put sequencing batch 1 in the training set and then added in patients from later batches to reach the target 50%/50% split. The sequencing lab decided which samples to include in batch 1 without any knowledge about the baseline characteristics, treatment or clinical outcomes of the patients. We then checked for imbalances, and it was found that RACE was not well distributed due to all Asian patients appearing in batch 1, and so we moved half of the Asian patients to the test set and replaced these spots in the training data with a random set of patients. As the analysis progressed (in the training subset of data), we decided to add in PBMC correction due to concern over germline/CHIP variants contaminating the ctDNA dataset, which reduced the number of patients to those with PBMC available for correction, giving a final *n* of 466 patients and a final split of 240/226 patients for train/test. The final training/test sets were well balanced in clinical features and survival outcomes as can be seen in Supplementary Table 2 and Extended Data Fig. 1f.

### Variant calling

Sequence data for the baseline plasma samples were processed by a cfDNA computational pipeline that corrected errors via the use of fragment barcodes previously described^35^. Short variants called by the bTMB assay were evaluated.

Sequence data processing and variant calling for on-treatment plasma and matched whole blood or PBMC samples were performed similarly to methods previously described^46^. In brief, reads were demultiplexed and fragment barcodes used to reduce errors, deduplicated and merged into consensus reads representing all information in the set of reads for each fragment. The consensus reads were then aligned to the reference genome.

For sensitively estimating on-treatment VAF, aligned consensus reads were postprocessed using proprietary software also developed by FMI. Each baseline variant was left- and right-justified to determine the locus of all possible overlapping reads. Those reads were then re-aligned to both the reference genome and the reference genome as modified by the presence of the variant. Striped Smith–Waterman alignment was used to score each of those two alignments, classifying each consensus read as either

- Supporting the presence of the variant (NRv)
- Supporting the absence of the variant (NRr)
- Has no discriminating value (equivocal) (NRe)

Equivocal, duplicate and low mapping quality reads were ignored.

VAF was estimated as NRv/(NRv + NRr).

To further refine the VAF, we assigned a classification by comparing it with the distribution of VAFs using the same method on a set of presumed normal samples that is samples whose baseline genomic profile had no nearby variant calls. The VAF classifications are:

- Positive—at least 2 variant supporting reads and VAF > maximum VAF found in presumed normal samples
- Negative—VAF < 0.95 quantiles of presumed normal VAFs
- Equivocal—neither of the above conditions is met.

Variants were called and classified in matched PBMCs using the same methodology described for ctDNA variant calling. Alterations that were classified as positive in PBMCs were presumed to be germline/CHIP and excluded from analyses.

### Statistics

To remove immortality bias^47^ when assessing the correlation between on-treatment measurements and PFS/OS, patients with events before the collection date were excluded from the analyses and PFS and OS time were recalculated from the on-treatment sample collection date. When utilizing both week 6 tumor response assessments and C3D1 ctDNA metrics, PFS/OS time was recalculated from the C3D1 date.

Landmark PFS and OS were compared between groups using a univariable Cox proportional-hazards model to estimate HR and log-rank test to report *P* values. Cox models used the ‘exact’ method for handling tied event times. *P* values reported in forest plots for multivariable Cox regression are using two-sided Wald test. The strength of the association between event time and a continuous predictor was measured by Harrell’s c-index, which indicates the overall rank concordance between event time and the predictor. Standard errors for the c-index were computed by assuming asymptotic normality^48^ and their *P* values test if an estimate is different from 0.5^49^. Two C indices were compared based on a U-statistic to test for whether one predictor is more concordant with the outcome than another (R package version 4.7-0. https://CRAN.R-project.org/package=Hmisc).

Descriptive statistics were used to summarize clinical characteristics and ctDNA metrics, including the mean, median and range for continuous variables and frequency and percentage for categorical variables. Association between ctDNA positivity and baseline prognostic factors was measured using a two-sided Wilcoxon Rank Sum test for numeric variables and Fisher’s Exact test (two-sided) for categorical variables. The association between continuous ctDNA metrics and radiographic treatment response categories was measured using a two-sided Wilcoxon rank sum test or Kruskal–Wallis rank sum test. Correlations between two continuous metrics depicted in scatterplots reported Pearson’s correlation.

Unless otherwise noted, all analyses combined patients across the three study arms and reported *P* values were two-sided and unadjusted for multiplicity or covariates. All statistical analyses were performed in R version 3.6 (https://www.R-project.org/). *P* values were reported for descriptive purposes and were unadjusted for multiple hypothesis testing.

### ctDNA feature derivation for predictive modeling in IMpower150 training/test and OAK validation data

Every ctDNA mutation had an associated AF reported by the assay at each time point, and in this study, the assay limit of quantitation (LOQ) and LOD were determined to be 0.5% and 0.1%, respectively (Extended Data Fig. 1b). Reported mutations with AF below the LOQ were censored to LOQ/2, and reported mutations with AF below the LOD were censored to LOQ/4.

The ctDNA analysis plan for the machine learning model was finalized before the development of the model. ctDNA levels (AF, MTM, AUC, etc.) were quantified using 23 different metrics measured for each time point (BL, C2D1, C3D1, C4D1 and C8D1), and the change in ctDNA relative to baseline was quantified using 55 different metrics for each on-treatment time point (C2D1, C3D1, C4D1 and C8D1).

Additional feature processing before running the ML model included handling missingness and interquartile range (IQR) normalization. If a patient in the ctDNA evaluable population has a record of a blood sample collection for a given visit with an associated date for that sample collection, then the patient is included in the landmark analysis for that visit. However, if the ctDNA data are missing despite the record of a blood sample collection (for example due to failing ctDNA assay QC), then ctDNA features were imputed using the population median of the feature for that visit. We considered this imputation for patients with ctDNA collected but QC-failing samples to be important because models included ctDNA features from multiple time points (that is C3D1 OS run included BL and C2D1 features as well) and so theoretically a patient with a QC-failing sample for C3D1 still may have C2 and/or BL ctDNA data informative for predicting survival time from C3D1. Final sample counts for each visit time point can be found in Extended Data Fig. 1g. Individual features were scaled by the IQR of that feature before running the machine learning model.

A complete list of ctDNA features can be found in Supplementary Table 3, along with the rank concordance (c-index) of each metric with landmark OS and PFS for each visit.

To test the validity of our ctDNA C3D1 OS model in an external cohort, we leveraged the availability of ctDNA data for *n* = 73 patients from the OAK clinical trial (NCT02008227). A continuous predictor was derived from the 5 ctDNA metrics measured by the Avenio panel and their coefficients which were used in the final C3D1 OS model. Note that for the feature ‘Number of pathogenic mutations detected at C3D1’, we considered Avenio mutations to be pathogenic when they were both nonsilent and present in COSMIC database (https://cancer.sanger.ac.uk/cosmic). OAK ctDNA features were processed as detailed for IMpower150 above, including censoring of small values, imputation for missing data and IQR normalization. Please note the censoring of small values occurred before feature derivation and used the same approach as described above for IMpower150 in which the LOD and LOQ were considered to be 0.1% and 0.5%, respectively. We applied the same thresholds identified in IMpower150 training data to identify high-risk mPD patients, low-risk mResp patients and intermediate-risk mSD patients.

### Training the ML model and choosing thresholds for mPD, mSD and mResp

At a visit, all ctDNA measurements collected from baseline up to the particular visit, among patients who are still at risk for PFS/OS, were used to associate with the rebaselined endpoint. All modeling was repeated with leave-one-out-cross-validation (LOOCV). Linear combinations of individual features were associated with landmark PFS and OS in an elastic network (R package glmnet v3.0-2) with an equal weight of lasso penalty and ridge penalty at each visit (alpha = 0.5). The optimal lasso penalty (lambda) was chosen for each LOOCV where nested cross-validation was repeated 10 times and the average of the lambda that minimizes the prediction error was used. Feature interaction was addressed in survival random forest modeling (R package randomForestSRC v 2.9.3) with default parameters, but no improvements in model performance were detected. Feature importance was assessed as the number of cross-validations a feature was retained, and by the ‘Gain’ metric as assessed by the average worsening statistic from the next-door analysis^40^ across LOOCV. Model performance, measured by c-index, was estimated after pooling all LOOCV predictions together to reconstruct the original training dataset. The final time point and endpoint were chosen to be C3D1 and OS due to numerically superior performance by c-index during LOOCV.

For model runs utilizing baseline clinical factors, the features included the following: ECOG score (0 or 1), age (continuous metric), number of metastatic sites (continuous metric), sex (M/F), history of tobacco use (y/n), PD-L1 high status (y/n) and SLD (of target lesions from radiographic assessment, a continuous metric). For the C3D1 model runs, we also included the week 6 radiographic tumor assessment data available, including week 6 SLD (continuous metric), difference in SLD between baseline and week 6 (continuous metric), and percent change in SLD between baseline and week 6 (continuous metric).

The final top features included in the ctDNA C3D1 OS model were features that were chosen in at least 50% of CV and with positive gain metric (5 features total) and can be found in Supplementary Table 5. The final C3D1 OS model was fit in the entire training set using these five features and coefficients can be found in Supplementary Table 6.

The threshold for the high-risk (mPD) group was chosen by visualizing different splits of the C3D1 OS model predictions for patients with week 6 SD and PR separately, choosing the optimal split within each and then taking the mean (Extended Data Fig. 4d) in the training dataset which corresponded to a numeric value of 0.298. The threshold for low-risk ctDNA responders (mResp) was chosen by finding the 32% percentile of the prediction scores, which corresponds to the proportion of patients who achieved durable (3 years) OS (Extended Data Fig. 4e). This 32% quantile of C3D1 OS model predictions corresponded to a numeric value of 0.036 in the training dataset (Extended Data Fig. 4e).

### Simulation of operation characteristics

To assess the utility of the ctDNA model in early clinical decision-making, with and without the radiographic endpoints, we performed operational characteristics analyses in simulated randomized phase 2 studies^42^.

Two routine endpoints used in early clinical developments are PFS and tumor response as assessed by the investigator according to RECIST criteria version 1.1. PFS is the time from randomization until tumor progression or death. We note that in phase 3 IMpower150 study patients have ~39.8 months of median follow-up; thus, these two endpoints are mature. In contrast, for this simulation, we are interested in early ctDNA and early PFS or tumor response signals observed within the first ~6 weeks of treatment initiation and whether these early endpoints can predict the outcome of the clinical trial (in the case of IMpower150, superior OS of treatment ABCP versus control BCP, or of treatment ACP versus control BCP).

The operation characteristics were assessed as follows: after running the final C3D1 OS model in the test dataset to obtain ctDNA model predictions, patients with predictions below the predefined threshold for mResp were identified. To characterize true go rates, we sampled 30 random patients (*n* = 2,000 simulations) from active arms (ABCP/ACP) arm and control (BCP) arm with replacement, which mimics those developmental settings where a Go decision is favorable. To characterize False Go Rates, we sampled two sets of 30 patients from the control arm, with one set as the standard of care treatment and the other as the new treatment, which mimics those development settings where a No-Go decision is favorable. In each simulated study, we compared the number of mResp patients between the treatment and control arms (Fisher’s exact test), as well as the distribution of PFS times (log-rank test), or number of radiographic response by RECIST (Fisher’s exact test). All *P* values were one-sided. When combining a ctDNA criterion with a RECIST criterion, the smaller one of the two *P* values is used for Go/No-Go decisions. For a single metric, the 15% (the desired False Go Rate) percentile of *P* values for an arm BCP versus BCP comparison was found (*n* = 2,000 simulations) and was considered the cutoff value for a Go decision for the arm ABCP/ACP versus BCP comparison. If the *P* value for ABCP-versus-BCP comparison is less than the cutoff value, then a Go decision is made, and the true go rate is the proportion of 2,000 *P* values less than the cutoff value. Under instantaneous enrollment scenario, all patients have the same length of follow. Under ramp-up enrollment, the clinical cutoff date is 14 days (expected ctDNA assay turnaround time) plus the C3D1 day of the last enrolled patient in a cohort of random samples. For simulation purposes, when selecting 30 patients, the actual enrollment of the chosen 30 must be within 12 months of each other.

### Reporting summary

Further information on research design is available in the Nature Portfolio Reporting Summary linked to this article.

## Online content

Any methods, additional references, Nature Portfolio reporting summaries, source data, extended data, supplementary information, acknowledgements, peer review information; details of author contributions and competing interests; and statements of data and code availability are available at 10.1038/s41591-023-02226-6.

## Supplementary information

Reporting Summary

Supplementary Tables Supplementary Tables 1–6.

## Data Availability

All clinical and ctDNA data for IMpower150 are deposited to the European Genome-Phenome Archive under accession number EGAS00001006703. Qualified researchers may request access to individual patient-level data through the clinical study data request platform (https://vivli.org/). Further details on Roche’s criteria for eligible studies are available at https://vivli.org/members/ourmembers. For further details on Roche’s Global Policy on the Sharing of Clinical Information and how to request access to related clinical study documents, see https://www.roche.com/research_and_development/who_we_are_how_we_work/clinical_trials/our_commitment_to_data_sharing.htm.

## Code availability

The documented code for the R statistical computing environment for analyses related to ctDNA in IMpower150 is deposited to the European Genome-Phenome Archive under accession number EGAS00001006703.
