# Supplementary material for: A longitudinal circulating tumor DNA-based model associated with survival in metastatic non-small-cell lung cancer
Source: Nat Med. 2023 Mar 16;29(4):859–68. doi: 10.1038/s41591-023-02226-6 (PMC10115641; doi:10.1038/s41591-023-02226-6)
Supplement: Supplementary file 1 — Reporting Summary [file 41591_2023_2226_MOESM1_ESM.pdf]

nature portfolio

Corresponding author(s): Zoe June Assaf
David Shames
Katja Schulze

Last updated by author(s): 01/11/2023

Reporting Summary

Nature Portfolio wishes to improve the reproducibility of the work that we publish. This form provides structure for consistency and transparency in reporting. For further information on Nature Portfolio policies, see our Editorial Policies and the Editorial Policy Checklist.

Statistics

For all statistical analyses, confirm that the following items are present in the figure legend, table legend, main text, or Methods section.

n/a	Confirmed	
☐	☒	The exact sample size (*n*) for each experimental group/condition, given as a discrete number and unit of measurement
☐	☒	A statement on whether measurements were taken from distinct samples or whether the same sample was measured repeatedly
☐	☒	The statistical test(s) used AND whether they are one- or two-sided<br>*Only common tests should be described solely by name; describe more complex techniques in the Methods section.*
☐	☒	A description of all covariates tested
☐	☒	A description of any assumptions or corrections, such as tests of normality and adjustment for multiple comparisons
☐	☒	A full description of the statistical parameters including central tendency (e.g. means) or other basic estimates (e.g. regression coefficient) AND variation (e.g. standard deviation) or associated estimates of uncertainty (e.g. confidence intervals)
☐	☒	For null hypothesis testing, the test statistic (e.g. *F*, *t*, *r*) with confidence intervals, effect sizes, degrees of freedom and *P* value noted<br>*Give P values as exact values whenever suitable.*
☒	☐	For Bayesian analysis, information on the choice of priors and Markov chain Monte Carlo settings
☐	☒	For hierarchical and complex designs, identification of the appropriate level for tests and full reporting of outcomes
☐	☒	Estimates of effect sizes (e.g. Cohen's *d*, Pearson's *r*), indicating how they were calculated

Our web collection on statistics for biologists contains articles on many of the points above.

Software and code

Policy information about availability of computer code

Data collection	No software used for data collection
Data analysis	All statistical analyses were performed in R (https://www.R-project.org/), version 3.6. R libraries included Hmisc (version 4.7), glmnet (v3.0-2), randomForestSRC (v 2.9.3). Code is deposited in a public repository (EGAS00001006703) and the link is in the Code Availability statement of the manuscript.

For manuscripts utilizing custom algorithms or software that are central to the research but not yet described in published literature, software must be made available to editors and reviewers. We strongly encourage code deposition in a community repository (e.g. GitHub). See the Nature Portfolio guidelines for submitting code & software for further information.

Data

Policy information about availability of data

All manuscripts must include a data availability statement. This statement should provide the following information, where applicable:

- Accession codes, unique identifiers, or web links for publicly available datasets
- A description of any restrictions on data availability
- For clinical datasets or third party data, please ensure that the statement adheres to our policy

Public datasets that were used during data processing included ExAC database (http://exac.broadinstitute.org/) and IDs from COSMIC database (https://cancer.sanger.ac.uk/cosmic).

The data and code required to reproduce results are deposited to the European Genome-Phenome Archive under accession number EGAS00001006703, and can be made available upon request. Qualified researchers may request access to individual patient-level data through the clinical study data request platform (https://vivli.org/). Further details on Roche's criteria for eligible studies are available at https://vivli.org/members/ourmembers. For further details on Roche's Global Policy on the Sharing of Clinical Information and how to request access to related clinical study documents, see https://www.roche.com/research_and_development/who_we_are_how_we_work/clinical_trials/our_commitment_to_data_sharing.htm.

Human research participants

Policy information about studies involving human research participants and Sex and Gender in Research.

Reporting on sex and gender

Gender was not collected in this study, and accordingly we do not use this term in the paper. Please refer to Extended Data Tables 1 and 2 for covariate-relevant population characteristics of the human research participants in the IMpower150 ctDNA study, which includes Sex.

Population characteristics

Patient metadata are included in the manuscript, including tables of population characteristics for both the full IMpower150 population and this retrospective exploratory ctDNA substudy reported in this manuscript (Supp Table ST1). Briefly, IMpower150 patients had stage IV or recurrent metastatic nonsquamous NSCLC for which they had not previously received chemotherapy, a baseline Eastern Cooperative Oncology Group (ECOG) performance-status score of 0 or 1, and tumor tissue available for biomarker testing and if they were eligible to receive bevacizumab; patients with any PD-L1 immunohistochemistry status were eligible. Please refer to Extended Data Tables 1 and 2 for covariate-relevant population characteristics of the human research participants in the IMpower150 ctDNA study, including baseline ECOG score, Age, Sex, Tobocco use, Race, Region, Number of metastatic sites, and PDL1 status.

Recruitment

In the IMpower150 study patients were eligible for inclusion in this retrospective exploratory ctDNA substudy reported in this manuscript if they had plasma samples available for ctDNA testing at both baseline and an early on-treatment timepoint (either cycle 2 day 1 or cycle 3 day 1), as well as PBMCs available in order to perform germline subtraction. This reduced the number of patients in this ctDNA substudy to 466. We expected a survivorship bias in this ctDNA-evaluable population due to our requirement for patients to have samples available after randomization, and while no strong PFS bias was found for ctDNA evaluable versus non-evaluable (HR=0.92 [0.82-1.05]), we did detect a survivorship bias for OS (HR=0.86 [0.75 – 0.99]) (Extended Data Figure ED1a). However, baseline characteristics were similar between the full IMpower150 and ctDNA evaluable population, including baseline ECOG, age, sex, race, region, among others (Supplementary Table ST1). We do not expect this OS survivorship to strongly impact our results, because this exploratory ctDNA substudy is concerned with building ctDNA-based models to predict overall survival, and typically patients who do not have evaluable on-treatment samples available are those who unfortunately had very rapid disease progression, and therefore for which a ctDNA model to predict survival is of limited utility.

Ethics oversight

The study was conducted in accordance with the International Conference on Harmonisation Good Clinical Practice guidelines and with the principles of the Declaration of Helsinki. All patients provided written informed consent, and the protocol was approved by independent ethics committees at each participating site. The study protocol is in the appendix of the primary clinical manuscript for IMpower150 (Socinski et al, NEJM 2018) and can also be found at https://clinicaltrials.gov/ct2/show/NCT02366143.
The are 161 institutes/organizations whose ethical committees approved the protocol, including:
[1] "Copernicus Group Independent Review Board"
[2] "Western Institutional Review Board"
[3] "Melbourne Health Human Research Ethics Committee"
[4] "Tasmania Health and Medical Human Research Ethics Committee"
[5] "Asociacion Benefica Prisma"
[6] "Comité Institucional de ética en Investigación Instituto Nacional de Enfermedades Neoplasicas"
[7] "Singhealth Centralised Institutional Review Board"
[8] "Institutional Review Board Kaohsiung Medical University Chung-Ho Memorial Hospital"
[9] "Institutional Review Board Taipei Veterans General Hospital"
[10] "Institutional Review Board"
[11] "Institutional Review Board of the Chi Mei Medical Center"
[12] "The Institutional Review Board of Taichung Veterans General Hospital"
[13] "Institutional Review Board of Tri-Service General Hospital"
[14] "Cheng-Hsin General Hospital Institutional Review Board"
[15] "Consejo de Evaluación Ética de Investigación en Salud - CoEIS"
[16] "Ministerio de Salud - Provincia de Río Negro"
[17] "Comité Independiente de Etica en investigación clínica \"Dr. Carlos A. Barclay"
[18] "Comité de ética en investigación Fundación Oncosalud"
[19] "Comisión Conjunta de Investigación en Salud (CCIS)"
[20] "Comité de Ética Centro de Oncología e Investigación Buenos Aires"
[21] "Comité de Ética Independiente - Fundación Sanatorio"
[22] "Institutional Review Board of Chang Gung Medical Foundation"
[23] "Ethikkommission für das Bundesland Salzburg"
[24] "The Ethics Committee for Clinical Trials of Medicinal Products"
[25] "Chesapeake Institutional Review Board"
[26] "St. Luke's Hospital & Health Network IRB"
[27] "St Charles Medical Center"
[28] "Saint Luke's Hospital Institutional Review Board"
[29] "Kantonale Ethikkommission Bern (KEK)"
[30] "Comite de Etica Cientifico del Servicio de Salud Metropolitano Norte"
[31] "Comitato Etico Area Vasta Nord Ovest presso Azienda Ospedaliero Universitaria Pisana di Pisa"

[32] "Comitato Etico Cardarelli-Santobono"
[33] "Comitato Etico San Martino IST 2"
[34] "Comitato Etico Lazio 1"
[35] "COMITATO ETICO DELL´UNIVERSITA´ CAMPUS BIO-MEDICO DI ROMA"
[36] "Medical Research Ethics Committees United"
[37] "CPP Sud-Méditerranée 2"
[38] "Sir Charles Gairdner Hospital HREC"
[39] "Bellberry Human Research Ethics Committee"
[40] "Cabrini Human Research Ethics Committee"
[41] "Comitato Etico Catania 1 presso A.O. Universitaria Policlinico Vittorio Emanuele di Catania"
[42] "Comissão de Ética para a Investigação Clínica - CEIC"
[43] "Ethikkommission an der Universität Regensburg"
[44] "Ethikkommission an der Universität Regensburg"
[45] "Lakeridge Health REB"
[46] "St. Joseph Mercy Health System Institutional Review Board #2 - Oncology Central IRB"
[47] "Mercy Saint Vincent Medical Center Institutional Review Board"
[48] "US Oncology Inc. Institutional Review Board"
[49] "Western Institutional Review Board (WIRB)"
[50] "CEIC de la Corporacion Sanitaria del Parc Tauli"
[51] "CEIC de Cantabria"
[52] "CEIC de Andalucia (CCEIBA)"
[53] "CEIC Hospital Universitario La Paz"
[54] "CEIC Fundación Jiménez Díaz"
[55] "CEIC Hospital Clínico Universitario de Valencia"
[56] "CEIC Hospital General Universitario Gregorio Marañon"
[57] "CEIC Hospital Clinico San Carlos"
[58] "CEIC Grupo Hospital de Madrid"
[59] "CEIC Parc de Salut Mar"
[60] "CEIC Islas Baleares (CEIC-IB)"
[61] "CEIC de Galicia (CAEI)"
[62] "CEIC Hospital Clinic de Barcelona"
[63] "Kaiser Permanente Southern California Institutional Review Board"
[64] "Research Ethics Committee of National Taiwan University Hospital"
[65] "Institutional Review Board of Chung Shan Medical University Hospital"
[66] "CHU de Liège - Comité d'Ethique"
[67] "CEQ of MI Kryvyi Rih Oncology Dispensary of Dnipropetrovsk Regional Council"
[68] "CEQ of Treatment and Prevention Institution Volyn Regional Oncology Dispensary"
[69] "CEQ of MI Dnipropetrovsk City Multifield Clinical Hospital #4 of Dnipropetrovsk Regional Council"
[70] "CEQ of Poltava Regional Clinical Oncology Dispensary of Poltava Regional Council"
[71] "Commission on Ethics Questions of Uzhgorod Central City Clinical Hospital"
[72] "Commission of Ethics Questions on the basis of the Chernivtsi Regional Clinical Oncology Dispensary"
[73] "CEQ of MI of Zaporizhzhia Regional Council Zaporizhzhia Regional Clinical Oncology Dispensary"
[74] "CEQ of Transcarpathian Regional Clinical Oncology Dispensary"
[75] "CEQ of SI Institute of Medical Radiology n.a. S.P. Hryhoriev of NAMS of Ukraine"
[76] "CEQ of Municipal Noncommercial Institution Regional Center of Oncology"
[77] "CEQ of Regional Municipal Institution Sumy Regional Clinical Oncology Dispensary"
[78] "Comitato Etico Azienda Ospedaliera Universitaria Maggiore della Carità"
[79] "Comitato Etico Dell Universita Cattolica del Sacro Cuore Policlinico Universitario Agostino Gemelli"
[80] "Comitê de Ética em Pesquisa em Seres Humanos da Faculdade de Medicina de São José do Rio Preto"
[81] "Comitê de Ética em Pesquisa em Seres Humanos do Hospital Socor"
[82] "Comitê de Ética em Pesquisa em Seres Humanos da Universidade de Ribeirão Preto (UNAERP)"
[83] "Comitê de Ética em Pesquisa em Seres Humanos da Irmandade da Santa Casa de Londrina"
[84] "Comitê de Ética em Pesquisa em Seres Humanos da Liga Norte Riograndense Contra o Câncer"
[85] "Comitê de Ética em Pesquisa Fundação Pio XII Hospital de Câncer de Barretos"
[86] "Comité de Ética en Investigación de la Facultad de Medicina y Hospital Universitario"
[87] "Missouri Baptist Medical Center Institutional Review Board"
[88] "University of California Irvine Institutional Review Board"
[89] "University of California"
[90] "Frederick Memorial Hospital Institutional Review Board"
[91] "Mercy Medical Center IRB"
[92] "University of Chicago Hospitals Institutional Review Board"
[93] "Mount Sinai Medical Center IRB"
[94] "Comité Provincial de Bioética - Ministerio de Salud de la Provincia de Santa Fé"
[95] "Comite de Etica Investigacion de la Clinica Bajío"
[96] "Comite de Etica en Investigacion de Mexico Centre for Clinical Research SA de CV"
[97] "Comitê de Ética em Pesquisa da Universidade de Caxias do Sul"
[98] "Comitê de Ética em Pesquisa do Hospital de Clínicas de Porto Alegre"
[99] "Comite de Etica em Pesquisa da Universidade Federal de Sao Paulo - Hospital Sao Paulo"
[100] "Comitê de Ética em Pesquisa - Hospital Mãe de Deus"
[101] "Comitê de Ética em Pesquisa da Fundação Antônio Prudente – Hospital do Câncer A. C. Camargo"
[102] "Ethics Committee for Multi-Centre Trials"
[103] "Ethics Committee at Clinical Oncology Dispensary"
[104] "Ethics Committee at City Clinical oncologic dispensary"
[105] "Ethics Committee at Russian Oncology Research Center n.a. N.N.Blokhin"
[106] "Ethics Committee at Moscow City Oncology Hospital #62 of Moscow Healthcare Department"
[107] "Ethics Committee at Volzhskiy regional clinical oncology dispensary #3"

[108] "CEQ of Ivano-Frankivsk Regional Oncology Dispensary"
[109] "CEIC Hospital Universitario Insular Materno-Infantil de Las Palmas"
[110] "CEIC Hospital Universitari Vall d'Hebron"
[111] "CEIC Hospital Universitario Ramon y Cajal"
[112] "CEIC Hospital Universitario 12 de Octubre"
[113] "Eticka komisia Presovskeho samospravneho kraja"
[114] "Eticka komisia Univerzitna nemocnica Bratislava"
[115] "Lithuanian Bioethics Committee"
[116] "CEIC Hospital Universitari de Bellvitge"
[117] "Eticka komisia pri Narodnom onkologickom ustave"
[118] "WIRB Copernicus Group"
[119] "Kaiser Permanente of Colorado Institutional Review Board"
[120] "Ethics Committee at Russian Medical Military Academy n.a. S.M.Kirov"
[121] "Comite de Etica en Investigacion del Instituto Regional de Enfermedades Neoplasicas"
[122] "National Cheng Kung University Hospital Human Experiment and Ethic Committee"
[123] "Houston Methodist Research Institute IRB"
[124] "Ingalls Memorial Hospital IRB"
[125] "Rush University Medical Center Institutional Review Board"
[126] "Mercy Health Springfield Communities Institutional Review Board"
[127] "Mayo Clinic Institutional Review Board"
[128] "Salus IRB"
[129] "Mackay Memorial Hospital Institutional Review Board"
[130] "Commission on Ethics Questions of Vinnytsya Regional Clinical Oncology Dispensary"
[131] "Comite Etico Cientifico Clinica Santa Maria"
[132] "BRANY IRB"
[133] "Yale University Human Research Protection Program"
[134] "Maimonides Med Ctr Institutional Review Board"
[135] "Scripps Health Institutional Review Board"
[136] "University of Texas Health Science Center San Antonio Institutional Review Board"
[137] "Park Nicollet Institute Institutional Review Board"
[138] "Eticka komisia NsP Sv. Jakuba"
[139] "Ethikkommission der Bayerischen Landesärztekammer"
[140] "CEQ of Kyiv City Clinical Oncological Center"
[141] "The Institutional Review Board of China Medical University Hospital"
[142] "Ethics Committee at Private Medical Institution \"Evromedservis\""
[143] "Comitato di Bioetica dell'AUSL 1 di Sassari"
[144] "Eticka komisia Onkologicky ustav sv. Alzbety"
[145] "Ethics Committee at Railway Clinical Hospital JSC RZhD"
[146] "Sault Area Hospital Research Ethics Board"
[147] "Toranomon Hospital and Toranomon Hospital Kajigaya IRB"
[148] "Kitasato University Sagamihara IRB"
[149] "Niigata Cancer Center Hospital IRB"
[150] "Kyoto University Hospital IRB"
[151] "Osaka City University Hospital IRB"
[152] "National Hosptial Organization Toneyama National Hospital IRB"
[153] "Wakayama Medical University IRB"
[154] "Kurume University IRB"
[155] "National Hospital Organization Kyushu Cancer Center"
[156] "Kanagawa Cardiovascular and Respiratory Center IRB"
[157] "National Hospital Organization Kyushu Medical Center IRB"
[158] "National Hospital Organization Shikoku Cancer Center IRB"
[159] "Miyagi Cancer Center IRB"
[160] "Kyorin University Hospital IRB"
[161] "Center Hospital of the National Center for Global Health and Medicine IRB"

Note that full information on the approval of the study protocol must also be provided in the manuscript.

Field-specific reporting

Please select the one below that is the best fit for your research. If you are not sure, read the appropriate sections before making your selection.

☒ Life sciences ☐ Behavioural & social sciences ☐ Ecological, evolutionary & environmental sciences

For a reference copy of the document with all sections, see nature.com/documents/nr-reporting-summary-flat.pdf

Life sciences study design

All studies must disclose on these points even when the disclosure is negative.

Sample size	Details on the IMpower150 study plan are published elsewhere. No sample size calculations were done for the ctDNA subsidy of IMpower150. The sample size was determined by the number of patients in IMpower150 (n=1201) which had baseline samples run on a 1.25 Mb ctDNA assay (n=1062), and those were subset to those patients with aliquots of C2D1 or C3D1 plasma available (n=910). 566 patients were then

	randomly chosen for the ctDNA subsidy, only 466 of which had PBMC available for CHIP/germline correction who were included in the final ctDNA analysis population.
Data exclusions	Patients were excluded if they did not have baseline plasma and C2D1/C3D1 and PBMC available. Please refer to Figure 1 for sample flow diagram.
Replication	Biomarkers measured in patient plasma samples were not replicated. Replication was not possible due to the limited patient samples available. The one experiment that had replication was during assay development in which 63 replicates were run to measure the concordance between the baseline assay and on-treatment assay. This experiment showed high concordance between the replicates (R2=0.9961; Supplementary Figure 1B right). No other experiments were replicated.
Randomization	Details on the original IMpower150 study plan including randomization has been published elsewhere and can also be found at https://clinicaltrials.gov/ct2/show/NCT02366143. For this exploratory retrospective ctDNA substudy reported in this manuscript, the 466 ctDNA-evaluable patients were split into a training cohort and a testing cohort for model development. The training and test sets were initially chosen based on sequencing batch for the set of 566 patients chosen for the ctDNA substudy (see Figure 1a), where we put sequencing batch1 in training set and then added in patients from later batches to reach the target 50%/50% split. The sequencing lab decided which samples to include in batch 1 without any knowledge about the baseline characteristics, treatment, or clinical outcomes of the patients. We then checked for imbalances and it was found that RACE was not well distributed due to all Asian patients appearing in batch1, and so we moved half of the Asian patients to the test set and replaced these spots in the training data with a random set of patients. As the analysis progressed (in the training subset of data) we decided to add in PBMC correction due to concern over germline/CHIP variants contaminating the ctDNA dataset, which reduced the number of patients to those with PBMC available for correction, giving a final n of 466 patients and a final split of 240/226 patients for train/test. The final training/test sets were well balanced in clinical features and survival outcomes as can be seen in Supplementary Table ST2 and Extended Data Figure ED1f.
Blinding	Details about blinding during arm allocation for the IMpower150 study itself can be found in the IMpower150 primary clinical manuscript or at https://clinicaltrials.gov/ct2/show/NCT02366143 . For this retrospective exploratory ctDNA substudy, our model development analysis was not initiated until after samples were allocated to either the training or testing subgroup. The allocation of training/testing was not performed blinded, as the goal was to ensure that the training and testing subgroups were similar in baseline characteristics. Sample collection was performed prior to this retrospective analysis, and so investigators were blinded during sample collection. Data was generated by a diagnostic company separate from the analysts who performed the model development, and so data generation was also performed by blinded individuals.

Reporting for specific materials, systems and methods

We require information from authors about some types of materials, experimental systems and methods used in many studies. Here, indicate whether each material, system or method listed is relevant to your study. If you are not sure if a list item applies to your research, read the appropriate section before selecting a response.

Materials & experimental systems

n/a	Involved in the study
☐	☒ Antibodies
☒	☐ Eukaryotic cell lines
☒	☐ Palaeontology and archaeology
☒	☐ Animals and other organisms
☐	☒ Clinical data
☒	☐ Dual use research of concern

Methods

n/a	Involved in the study
☒	☐ ChIP-seq
☒	☐ Flow cytometry
☒	☐ MRI-based neuroimaging

Antibodies

Antibodies used	PD-L1 expression on tumor cells or tumor-infiltrating immune cells was analyzed in archival or freshly collected tumor tissue (or both) with the use of a PD-L1 immunohistochemistry assay (Ventana Medical Systems; clone SP142; catalog number N/A; pre-dilute ready to use antibody product at 36ug/5mL)
Validation	Ventana PD-L1 (SP142) assay validation can be found at the following link: https://www.accessdata.fda.gov/cdrh_docs/pdf16/p160002c.pdf

Clinical data

Policy information about clinical studies

All manuscripts should comply with the ICMJE guidelines for publication of clinical research and a completed CONSORT checklist must be included with all submissions.

Clinical trial registration	NCT02366143
Study protocol	The protocol was published with the primary clinical manuscript and can be found here: https://www.nejm.org/doi/suppl/10.1056/NEJMoa1716948/suppl_file/nejmoa1716948_protocol.pdf

Data collection

Details of clinical data collection can be found in primary clinical manuscript for IMpower150. Briefly, IMpower150 data was collected between March 2015 and December 2019, patients from 26 countries or regions were enrolled at 240 sites. The list of 240 sites is the following:
United States, Arizona
Ironwood Cancer & Research Centers
Chandler, Arizona, United States, 85224
Arizona Oncology Associates
Flagstaff, Arizona, United States, 86001
United States, California
Southern CA Permanente Med Grp
Bellflower, California, United States
Marin Cancer Care Inc
Greenbrae, California, United States, 94904
Scripps Health
La Jolla, California, United States, 92037
Chao Family Comprehensive Cancer Center UCI
Orange, California, United States, 92868
United States, Colorado
Rocky Mountain Cancer Center
Denver, Colorado, United States, 80218
Kaiser Permanente
Lonetree, Colorado, United States, 80124
United States, Connecticut
Danbury Hospital
Danbury, Connecticut, United States, 06810
Yale Cancer Center
New Haven, Connecticut, United States, 06520
United States, Florida
Holy Cross Hospital Inc
Fort Lauderdale, Florida, United States, 33308
Cancer Specialists of North Florida - Baptist South
Jacksonville, Florida, United States, 32258
Mount Sinai Medical Center
Miami Beach, Florida, United States, 33140
Hematology Oncology Associates of the Treasure Coast
Port Saint Lucie, Florida, United States, 34952
United States, Georgia
Piedmont Cancer Institute, PC
Atlanta, Georgia, United States, 30318
United States, Illinois
Rush University Medical Center
Chicago, Illinois, United States, 60612
Univ of Chicago
Chicago, Illinois, United States, 60637
Ingalls Memorial Hospital
Harvey, Illinois, United States, 60426
Oncology Specialists, S.C.
Park Ridge, Illinois, United States, 60068
United States, Iowa
Hematology-Oncology; Associates of the Quad Cities
Bettendorf, Iowa, United States, 52722
United States, Kentucky
Norton Cancer Institute
Louisville, Kentucky, United States, 40202
United States, Maine
New England Cancer Specialists
Scarborough, Maine, United States, 04074
United States, Maryland
Mercy Medical Center
Baltimore, Maryland, United States, 21202
Regional Cancer Care Associates
Bethesda, Maryland, United States, 20817
Maryland Oncology Hematology, P.A.
Columbia, Maryland, United States, 21044
United States, Michigan
St. Joseph Mercy Health System
Ann Arbor, Michigan, United States, 48106
United States, Minnesota
St. Luke's Regional Cancer Center
Duluth, Minnesota, United States, 55805
Park Nicolett - Frauenshuh Cancer Center
Saint Louis Park, Minnesota, United States, 55426
United States, Missouri
St. Luke's Cancer Institute
Kansas City, Missouri, United States, 64111

Missouri Baptist Medical Center
Saint Louis, Missouri, United States, 63131
United States, Montana
Billings Clinic
Billings, Montana, United States, 59102
Montana Cancer Specialists
Missoula, Montana, United States, 59802
United States, Nevada
Comprehensive Cancer Centers of Nevada
Henderson, Nevada, United States, 89014
United States, New Jersey
Summit Medical Group
Berkeley Heights, New Jersey, United States, 07922
Valley Hospital; Oncology Research
Paramus, New Jersey, United States, 07652
Regional Cancer Care Associates LLC
Sewell, New Jersey, United States, 08080
United States, New York
Montefiore Medical Center
Bronx, New York, United States, 10467
Maimonides Medical Center
Brooklyn, New York, United States, 11219
United States, North Carolina
First Health of the Carolinas
Pinehurst, North Carolina, United States, 28374
United States, Ohio
University of Cincinnati
Cincinnati, Ohio, United States, 45203-0542
Mercy St Anne Hospital
Toledo, Ohio, United States, 43623
United States, Oregon
Bend Memorial Clinic
Bend, Oregon, United States, 97701
St. Charles Medical Center Bend; Cancer Care Of The Cascades
Bend, Oregon, United States, 97701
Willamette Valley Cancer Insitute and Research Center
Springfield, Oregon, United States, 97477
United States, Pennsylvania
St. Luke's Cancer Care Associates
Bethlehem, Pennsylvania, United States, 18015
Allegheny Cancer Center
Pittsburgh, Pennsylvania, United States, 15212
Univ of Pittsburgh Medical Ctr
Pittsburgh, Pennsylvania, United States, 15232
United States, Tennessee
West Clinic
Germantown, Tennessee, United States, 38138
Tennessee Cancer Specialists
Knoxville, Tennessee, United States, 37920
United States, Texas
Houston Methodist Cancer Center
Houston, Texas, United States, 77030
Longview Cancer Center
Longview, Texas, United States, 75601
University of Texas Health Science Center at San Antonio
San Antonio, Texas, United States, 78229
United States, Virginia
Virginia Cancer Specialists, PC
Fairfax, Virginia, United States, 22031
Virginia Oncology Associates
Norfolk, Virginia, United States, 23502
Virginia Cancer Institute
Richmond, Virginia, United States, 23226
Blue Ridge Cancer Care
Roanoke, Virginia, United States, 24014
United States, Washington
MultiCare Regional Cancer Center - Auburn
Auburn, Washington, United States, 98002-4117
Providence Regional Cancer Partnership
Everett, Washington, United States, 98201
Virginia Mason Medical Center
Seattle, Washington, United States, 98101
Medical Oncology Associates
Spokane, Washington, United States, 99208
United States, West Virginia
West Virginia University; Mary Babb Randolph Can Ctr

Morgantown, West Virginia, United States, 26506
Argentina
Centro Medico Austral
Buenos Aires, Argentina, 1019
Fundación CENIT para la Investigación en Neurociencias
Buenos Aires, Argentina, C1125ABD
Sanatorio Allende
Cordoba, Argentina, X5000JHQ
Centro Oncologico Riojano Integral (CORI)
La Rioja, Argentina, F5300COE
Hospital Provincial del Centenario
Rosario, Argentina, 2000
Fundacion Koriza
Santa Rosa, Argentina, 6300
Centro de Investigacion; Clinica - Clinica Viedma S.A.
Viedma, Argentina, R8500ACE
Australia, New South Wales
Chris O'Brien Lifehouse
Camperdown, New South Wales, Australia, 2050
Concord Repatriation General Hospital
Concord, New South Wales, Australia, 2139
Nepean Cancer Care Centre
Sydney, New South Wales, Australia, 2747
Australia, Queensland
Prince Charles Hospital; Department of Medical Oncology
Chermside, Queensland, Australia, 4032
Townsville Hospital
Townsville, Queensland, Australia, 4810
Princess Alexandra Hospital
Woolloongabba, Queensland, Australia, 4102
Australia, South Australia
Royal Adelaide Hospital
Adelaide, South Australia, Australia, 5000
Adelaide Cancer Centre
Kurralta Park, South Australia, Australia, 5037
Australia, Tasmania
Royal Hobart Hospital
Hobart, Tasmania, Australia, 7000
Launceston General Hospital
Launceston, Tasmania, Australia, 7250
Australia, Victoria
Frankston Hospital
Frankston, Victoria, Australia, 3199
Austin Health
Heidelberg, Victoria, Australia, 3084
Cabrini Hospital Malvern
Malvern, Victoria, Australia, 3144
The Alfred Hospital
Prahan, Victoria, Australia, 3181
Sunshine Hospital
St Albans, Victoria, Australia, 3021
Australia, Western Australia
Sir Charles Gairdner Hospital
Nedlands, Western Australia, Australia, 6009
Austria
Paracelsus Medizinische Privatuniversität
Salzburg, Austria, 5020
Klinikum Wels-Grieskirchen
Wels, Austria, 4600
Belgium
CHU de Liège
Liège, Belgium, 4000
Clinique Ste-Elisabeth
Namur, Belgium, 5000
Brazil
CETUS Hospital Dia Oncologia
Uberaba, MG, Brazil, 38082-049
Instituto Do Cancer Delondrina_X; Unidade De Pesquisa Clinica
Londrina, PR, Brazil, 86 015 520
Liga Norte Riograndense Contra O Câncer
Natal, RN, Brazil, 59040150
IPCEM; Instituto de Pesquisa de Estudos Multicêntricos
Caxias do Sul, RS, Brazil, 95070-560
Hospital Mae de Deus
Porto Alegre, RS, Brazil, 90470-340
Hospital de Cancer de Barretos

Barretos, SP, Brazil, 14784-400
Instituto Ribeirãopretano de Combate Ao Câncer; Centro Especializado De Oncologia
Ribeirão Preto, SP, Brazil, 14015-130
Hospital de Base de Sao Jose do Rio Preto
Sao Jose do Rio Preto, SP, Brazil, 15090-000
Hospital A. C. Camargo; Oncologia
Sao Paulo, SP, Brazil, 01509-010
Bulgaria
Multiprofile Hospital for Active Treatment Central Onco Hospital OOD
Plovdiv, Bulgaria, 4000
MHAT Serdika, EOOD
Sofia, Bulgaria, 1303
Canada, Ontario
Lakeridge Health Center
Oshawa, Ontario, Canada, L1J 2J2
Chile
Clinica Santa Maria
Santiago, Chile, 0
Health & Care SPA
Santiago, Chile, 7500006
Sociedad de Investigaciones Medicas Ltda (SIM)
Temuco, Chile, 4810469
France
Institut Bergonié Centre Régional de Lutte Contre Le Cancer de Bordeaux Et Sud Ouest
Bordeaux, France, 33076
CHU de Grenoble
Grenoble, France, 38043
Centre Jean Bernard Clinique Victor Hugo
Le Mans, France, 72015
Hôpital Saint Joseph
Marseille, France, 13008
Hopital Nord AP-HM
Marseille, France, 13015
Hôpital Européen Georges Pompidou
Paris, France, 75908
CHU de Bordeaux
Pessac, France, 33600
Service de Pneumologie Centre Hospitalier Régional La Réunion Site Felix Guyon
Saint Denis Cedex, France, 97405
CH de Saint Quentin
Saint Quentin, France, 2100
Centre Hospitalier Intercommunal Toulon - La Seyne sur Mer
Toulon, France, 83000
Hôpital d'Instruction des Armées de Sainte Anne; Service de Pneumologie
Toulon, France, 83000
Hôpital Larrey;Université Paul Sabatier
Toulouse, France, 31059
Germany
Zentralklinikum Augsburg
Augsburg, Germany, 86156
Helios Klinikum Emil von Behring GmbH
Berlin, Germany, 14165
Ev.Krankenhaus Bielefeld gGmbH; Klinik für Innere Medizin und Geriatrie
Bielefeld, Germany, 33611
Augusta Kranken-Anstalt gGmbH
Bochum, Germany, 44791
Universitätsklinikum "Carl Gustav Carus" der Technischen Universität Dresden
Dresden, Germany, 01307
St. Elisabethen Krankenhaus
Frankfurt am Main, Germany, 60487
LungenClinic Großhansdorf GmbH
Großhansdorf, Germany, 22927
Krankenhaus Martha-Maria; Halle-Dolau gGmbH
Halle, Germany, 06120
Asklepios Klinik Harburg
Hamburg, Germany, 21075
Lungenklinik Hemer
Hemer, Germany, 58675
Universität Des Saarlandes; Klinik für Innere Medizin V
Homburg, Germany, 66421
Kliniken der Stadt Koln gGmbH; Lungenklinik Onkologische Ambulanz
Koln, Germany, 51109
Klinik Loewenstein gGmbH; Onk & Pal
Loewenstein, Germany, 74245
Klinikum Bogenhausen
München, Germany, 81925

Krankenhaus Barmherzige Bruder Regensburg
Regensburg, Germany, 93049
Klinikum der Universität Regensburg
Regensburg, Germany, 93053
Stiftung Mathias-Spital Rheine
Rheine, Germany, 48431
Italy
AORN A Cardarelli
Napoli, Campania, Italy, 80131
Policlinico Universitario Campus Biomedico; Uoc Oncologia Medica
Roma, Lazio, Italy, 00128
Azienda Ospedaliera San Camillo Forlanini
Roma, Lazio, Italy, 00152
Università Cattolica Del S Cuore
Roma, Lazio, Italy, 00168
Istituto Nazionale per la Ricerca sul Cancro di Genova
Genova, Liguria, Italy, 16132
ASL 3 Genovese; DSM
Genova, Liguria, Italy, 16147
A.O.U. Maggiore della Carità
Novara, Piemonte, Italy, 28100
Azienda Unita Sanitaria Locale N1 Sassari; Unita Operativa Di Oncologia Medica
Sassari, Sardegna, Italy, 07100
Policlinico Vittorio Emanuele
Catania, Sicilia, Italy, 95123
Ospedale Versilia
Lido Di Camaiore, Toscana, Italy, 55043
Ospedale Civile - Livorno
Livorno, Toscana, Italy, 57124
Japan
National Hospital Organization Shikoku Cancer Center
Ehime, Japan, 791-0280
National Hospital Organization Kyushu Medical Center
Fukuoka, Japan, 810-8563
NHO Kyushu Cancer Center
Fukuoka, Japan, 811-1395
Kurume University Hospital
Fukuoka, Japan, 830-0011
Kanagawa Cardiovascular and Respiratory Center
Kanagawa, Japan, 236-0051
Kitasato University Hospital
Kanagawa, Japan, 252-0375
Kyoto University Hospital
Kyoto, Japan, 606-8507
Miyagi Cancer Center
Miyagi, Japan, 981-1293
Niigata Cancer Center Hospital
Niigata, Japan, 951-8566
Osaka City University Hospital
Osaka, Japan, 545-8586
National Hospital Organization Osaka Toneyama Medical Center
Osaka, Japan, 560-8552
Toranomon Hospital
Tokyo, Japan, 105-8470
Center Hospital of the National Center for Global Health and Medicine
Tokyo, Japan, 162-0052
Kyorin University Hospital
Tokyo, Japan, 181-8611
Wakayama Medical University Hospital
Wakayama, Japan, 641-8510
Latvia
Riga East Clinical University Hospital Latvian Oncology Centre
Riga, Latvia, LV-1079
Pauls Stradins Clinical University Hospital
Rīga, Latvia, LV-1002
Lithuania
National Cancer Institute
Vilnius, Lithuania, 08660
Mexico
Centro Universitario Contra El Cancer
Monterrey, Mexico, 64020
Cancerologia de Queretaro
Queretaro, Mexico, 76090
Centro Hemato Oncologico Privado; Oncologia
Toluca, Mexico, 50080
Netherlands

Jeroen Bosch Ziekenhuis
'S Hertogenbosch, Netherlands, 5223 GZ
Amsterdam UMC Location VUMC
Amsterdam, Netherlands, 1081 HV
Amphia Ziekenhuis; Afdeling Longziekten
Breda, Netherlands, 4818 CK
Ziekenhuis Gelderse Vallei
EDE, Netherlands, 6716 RP
Tergooiziekenhuizen
Hilversum, Netherlands, 1201 DA
Spaarne Gasthuis; Spaarne Ziekenhuis
Hoofddorp, Netherlands, 2134 TM
Maastricht University Medical Center
Maastricht, Netherlands, 6229 HX
St. Antonius Ziekenhuis; R&D Long
Nieuwegein, Netherlands, 3435 CM
Erasmus MC; Afdeling Longziekten
Rotterdam, Netherlands, 3015 GD
Maasstad ziekenhuis
Rotterdam, Netherlands, 3079 DZ
Universitair Medisch Centrum Utrecht
Utrecht, Netherlands, 3584 CX
Gelre Ziekenhuizen, Zutphen
Zutphen, Netherlands, 7207 AE
Peru
Centro Medico Monte Carmelo
Arequipa, Peru, 04001
Centro Especializado de Enfermedades Neoplásicas
Arequipa, Peru
Instituto Nacional de Enfermedades Neoplasicas
Lima, Peru, Lima 34
Portugal
Centro Hospitalar E Universitário de Coimbra EPE
Coimbra, Portugal, 3000-602
Instituto Portugues Oncologia de Lisboa Francisco Gentil EPE
Lisboa, Portugal, 1099-023
Hospital Pulido Valente; Servico de Pneumologia
Lisboa, Portugal, 1796-001
Centro Hospitalar do Porto - Hospital de Santo António
Porto, Portugal, 4099-001
Instituto Portugues de Oncologia Do Porto Francisco Gentil Epe
Porto, Portugal, 4200-072
Hospital de Sao Joao; Servico de Pneumologia
Porto, Portugal, 4200
Russian Federation
Moscow City Oncology Hospital #62
Moscovskaya Oblast, Moskovskaja Oblast, Russian Federation, 143423
Russian Oncology Research Center n.a. N.N. Blokhin
Moscow, Russian Federation, 115478
Clinical Oncology Dispensary
Omsk, Russian Federation, 644013
Evromedservis LCC
Pushkin, Russian Federation, 196603
City Clinical Oncology Dispensary
Saint-Petersburg, Russian Federation, 197022
Singapore
National Cancer Centre; Medical Oncology
Singapore, Singapore, 169610
Slovakia
Univerzitna nemocnica Bratislava
Bratislava, Slovakia, 813 69
Narodny onkologicky ustav
Bratislava, Slovakia, 833 10
POKO Poprad s.r.o.
Poprad, Slovakia, 058 01
Spain
Instituto Catalan de Oncologia de Hospitalet (ICO); Servicio de Farmacia
L'Hospitalet de Llobregat, Barcelona, Spain, 08908
Corporacio Sanitaria Parc Tauli; Servicio de Oncologia
Sabadell, Barcelona, Spain, 8208
Hospital Universitario Marques de Valdecilla
Santander, Cantabria, Spain, 39008
Hospital Universitario Son Espases
Palma De Mallorca, Islas Baleares, Spain, 07014
Hospital Universitario Insular de Gran Canaria
Las Palmas de Gran Canaria, LAS Palmas, Spain, 35016

Complejo Hospitalario U. de Ourense
Ourense, Orense, Spain, 32005
Hospital Lluis Alcanyis De Xativa
Xativa, Valencia, Spain, 46800
Hospital del Mar
Barcelona, Spain, 08003
Hospital Univ Vall d'Hebron; Servicio de Oncologia
Barcelona, Spain, 08035
Hospital Clinic de Barcelona
Barcelona, Spain, 08036
Hospital Universitario Reina Sofia
Cordoba, Spain, 14004
Hospital Lucus Augusti; Servicio de Oncologia
Lugo, Spain, 27003
Hospital General Universitario Gregorio Marañon; Servicio de Oncologia
Madrid, Spain, 28007
Hospital Universitario La Paz
Madrid, Spain, 280146
Hospital Ramon y Cajal; Servicio de Oncologia
Madrid, Spain, 28034
Hospital Clinico San Carlos; Servicio de Oncologia
Madrid, Spain, 28040
Hospital Universitario Fundación Jimenez Díaz
Madrid, Spain, 28040
Hospital Universitario 12 de Octubre
Madrid, Spain, 28041
Hospital Universitario HM Sanchinarro-CIOCC
Madrid, Spain, 28050
Hospital Clinico Universitario de Valencia
Valencia, Spain, 46010
Switzerland
Kantonsspital Baselland
Bruderholz, Switzerland, 4101
Luzerner Kantonsspital Sursee
Luzern, Switzerland, 6000
Kantonsspital St. Gallen; Onkologie/Hämatologie
St. Gallen, Switzerland, 9007
Taiwan
Changhua Christian Hospital; Hematology-Oncology
Changhua, Taiwan, 500
Kaohsiung Medical University Hospital; Department of Urology
Kaohsiung City, Taiwan, 807
Chi Mei Medical Center Liou Ying Campus
Liuying Township, Taiwan, 736
Chang Gung Memorial Hospital Chiayi
Putzu, Taiwan, 613
National Cheng Kung Univ Hosp
Tainan, Taiwan, 00704
National Taiwan Uni Hospital
Taipei City, Taiwan, 10041
Cheng Hsin General Hospital
Taipei, Taiwan, 112
Tri-Service General Hospital
Taipei, Taiwan, 11490
Chang Gung Medical Foundation Linkou Branch
Taoyuan City, Taiwan, 333
Taichung Veterans General Hospital
Xitun Dist., Taiwan, 40705
Ukraine
ME Bukovinian Clinical Oncology Center
Chernivtsi, Chernihiv Governorate, Ukraine, 58013
Municipal Institution City Clinical Hospital #4 of Dnipro City Council - PPDS; Dept of Chemotherapy
Dnipropetrovsk, Katerynoslav Governorate, Ukraine, 49102
Uzhgorod Central City Clinical Hospital
Uzhhorod, Katerynoslav Governorate, Ukraine, 88000
MNPE Zaporizhzhia Regional Antitumor Center ZRC
Zaporizhzhia, Katerynoslav Governorate, Ukraine, 69040
Communal Non profit Enterprise Regional Center of Oncology; Oncosurgical dept of thoracic organs
Kharkiv, Kharkiv Governorate, Ukraine, 61070
MNPE Transcarpathian Antitumor Center of the Transcarpathian Regional Council; Chemotherapy Dept
Uzhhorod, Kherson Governorate, Ukraine, 88014
Municipal Institution SubCarpathian Clinical Oncological Centre; Surgical department#2
Ivano-Frankivsk, KIEV Governorate, Ukraine, 76018
Communal Nonprofit Enterprise Podilsky Regional Center Of Oncology OfTheVinnytsia Regional Council
Vinnytsia, KIEV Governorate, Ukraine, 21029
SI Institute of Medical Radiology n.a. S.P. Hryhoriev of NAMS of Ukraine

Kharkiv, Ukraine, 61024
ME Kryviy Rih Oncology Dispensary of Dnipropetrovs'k Regional Council; Chemotherapy Department
Kryvyi Rih, Ukraine, 50048
Kyiv City Clinical Oncological Center
Kyiv, Ukraine, 03115
Poltava Regional Clinical Oncology Dispensary of Poltava Regional Council; Thoracic department
Poltava, Ukraine, 36011
Regional Municipal Institution Sumy Regional Clinical Oncology Dispensary
Sumy, Ukraine, 40005

Outcomes

The two primary end points of IMpower150 were progression-free survival (as assessed by investigators according to RECIST criteria) both among patients in the intention-to-treat population who had a wild-type genotype (WT population; patients with EGFR or ALK genomic alterations were excluded) and among patients in the WT population who had high expression of an effector T-cell (Teff) gene signature in the tumor (Teff-high WT population) and overall survival in the WT population. The ctDNA substudy also used the Overall Survival and radiographic response as assessed by RECIST v1.1. Additional details on the IMpower150 study can be found in the primary clinical manuscript.
